# Calcium binding of the antifungal protein PAF: Structure, dynamics and function aspects by NMR and MD simulations

**DOI:** 10.1371/journal.pone.0204825

**Published:** 2018-10-15

**Authors:** Ádám Fizil, Christoph Sonderegger, András Czajlik, Attila Fekete, István Komáromi, Dorottya Hajdu, Florentine Marx, Gyula Batta

**Affiliations:** 1 Department of Organic Chemistry, Faculty of Science and Technology, University of Debrecen, Debrecen, Hungary; 2 Division of Molecular Biology, Biocenter, Medical University of Innsbruck, Innsbruck, Austria; 3 Division of Clinical Laboratory Science, Department of Laboratory Medicine, Faculty of Medicine, University of Debrecen, Debrecen, Hungary; Russian Academy of Medical Sciences, RUSSIAN FEDERATION

## Abstract

Calcium ions (Ca^2+^) play an important role in the toxicity of the cysteine-rich and cationic antifungal protein PAF from *Penicillium chrysogenum*: high extracellular Ca^2+^ levels reduce the toxicity of PAF in the sensitive model fungus *Neurospora crassa* in a concentration dependent way. However, little is known about the mechanistic details of the Ca^2+^ ion impact and the Ca^2+^ binding capabilities of PAF outside the fungal cell, which might be the reason for the activity loss. Using nuclear magnetic resonance (NMR), isothermal titration calorimetry and molecular dynamics (MD) simulations we demonstrated that PAF weakly, but specifically binds Ca^2+^ ions. MD simulations of PAF predicted one major Ca^2+^ binding site at the C-terminus involving Asp53 and Asp55, while Asp19 was considered as putative Ca^2+^ binding site. The exchange of Asp19 to serine had little impact on the Ca^2+^ binding, however caused the loss of antifungal activity, as was shown in our recent study. Now we replaced the C-terminal aspartates and expressed the serine variant PAF^D53S/D55S^. The specific Ca^2+^ binding affinity of PAF^D53S/D55S^ decreased significantly if compared to PAF, whereas the antifungal activity was retained. To understand more details of Ca^2+^ interactions, we investigated the NMR and MD structure/dynamics of the free and Ca^2+^-bound PAF and PAF^D53S/D55S^. Though we found some differences between these protein variants and the Ca^2+^ complexes, these effects cannot explain the observed Ca^2+^ influence. In conclusion, PAF binds Ca^2+^ ions selectively at the C-terminus; however, this Ca^2+^ binding does not seem to play a direct role in the previously documented modulation of the antifungal activity of PAF.

## Introduction

The small antifungal protein PAF is secreted by the filamentous ascomycete *Penicillium chrysogenum*. Since PAF shows no toxic effect on mammalian cells it represents a promising bio-molecule[1] to prevent the growth of human and plant pathogenic fungi[2, 3]. The structures of PAF and related proteins generally consist of five antiparallel β-strands and the β-barrel tertiary structures are stabilized by three or four disulphide bonds that results in extreme temperature, pH and even protease stability[1, 4, 5]. Therefore, they may be excellent examples for rational drug design to develop new antifungal molecules. In order to understand the relationship between their biological activity and structural properties we have examined several of these proteins recently, especially PAF from the β-lactam antibiotics producer *Penicillium chrysogenum*[1]. The solution structure of PAF was determined and its interesting dynamical behaviour was described previously[5, 6]. In the course of our intensive studies addressing the mechanistic function of this protein, we showed that PAF acts in a very complex way including activities at the outer fungal cell layers and also inside the target cell[1, 7, 8]. The perturbation of intracellular calcium (Ca^2+^) homeostasis was found to be closely associated with its toxic function[9, 10]. Ca^2+^ plays an essential role in all organisms in intracellular signalling and acts as an important second messenger in numerous signal transduction pathways that regulate various cellular responses. In the PAF-sensitive model fungi *Neurospora crassa* and *Aspergillus niger*, PAF elicits an immediate and sustained elevation of the cytosolic free Ca^2+^ [Ca^2+^]_c_ concentration that is triggered by an influx of extracellular Ca^2+^ ions. In consequence, the PAF treated fungal cells are disturbed in polar growth and cell proliferation, and show apoptotic markers[1, 7, 9]. There is increasing evidence that the perturbation of the fungal [Ca^2+^]_c_ homeostasis is a common mechanism that is also responsible for the growth inhibitory activity of other antifungal proteins, e.g. the antifungal protein AFP_NN5353_ of *Aspergillus giganteus*[11], several plant defensins[12] and the antifungal hexapeptide RKKWFW (PAF26)[13].

Importantly, the supplementation of the fungal growth medium with CaCl_2_ diminishes the detrimental effects of PAF in a concentration dependent way by disturbing the interaction of PAF with the target cell, preventing the elevation of [Ca^2+^]_c_ and counteracting cell death[8, 9]. The reason for this neutralizing effect of extracellular Ca^2+^ ions on the antifungal action of PAF outside the fungal cell is still unclear.

In this study, we combined a molecular biology approach with structural analyses and functional tests to understand the role of extracellular Ca^2+^ in the mode of action of PAF. In particular, we addressed the hypothesis that this process involves a direct interaction of Ca^2+^ with this antifungal protein. Molecular dynamics (MD) simulations indicated that the preferred Ca^2+^ binding site is formed by the negatively charged C-terminal carboxylate group, together with the carboxylate groups of the C-terminally located Asp53 and Asp55. We examined the roles of these critical amino acid (aa) residues in Ca^2+^ binding affinity, 3D solution structure and the antifungal toxicity by comparing the recombinant expressed PAF^D53S/D55S^, in which the Asp residues were substituted for serines (Ser), with PAF. We also analysed the recently described single mutant PAF^D19S^, in which the less probable Ca^2+^ binding residue Asp19 was substituted for a Ser[8]. Finally, structural and biophysical tests were performed for the Ca^2+^-bound PAF (PAF-Ca^2+^).

## Materials and methods

### Strains and growth conditions

Fungal strains used in this study are listed in Table A in S1 File. *P*. *chrysogenum* shaking cultures were grown as described before[14]. In brief, 200 mL *P*. *chrysogenum* minimal medium (*Pc*MM) was inoculated with 2x10^8^ conidia and grown for 72 h at 25 °C under continuous shaking. Isotopic ^15^N-labeling of proteins for nuclear magnetic resonance (NMR) analysis was performed by replacing the nitrogen source with 0.3% Na^15^NO_3_ (Euriso-Top)[14]. For antifungal activity assays, *N*. *crassa* was used as a PAF-sensitive model organism and cultivated in 5-fold diluted Vogel’s medium[15] at 25 °C. Spores were generated from surface cultures cultivated on Vogel’s agar at 37 °C for 24 h under continuous light.

### Generation of PAF and PAF variants

Recombinant PAF and PAF^D19S^ were generated as described previously[8]. Site-directed mutagenesis in the *paf* gene was applied to prepare the expression plasmid pSK275p*af* ^D53S/D55S^ for PAF^D53S/D55S^ production. The primers used are listed in Table B in S1 File. After verifying the correct mutation of the *paf* nucleotide sequence by Sanger sequencing (Eurofins/MWG Operon), the plasmid pSK275*paf* ^D53S/D55S^ was transformed into a *P*. *chrysogenum* Δ*paf* strain[16] as previously published[14]. The expression of PAF^D53S/D55S^ in liquid culture and the verification of the correct aa exchanges by electro spray ionization mass spectrometry (ESI-MS) at the Protein MicroAnalysis Facility (Medical University of Innsbruck) were performed as described[8, 14].

### Antifungal activity assays

Fungal growth inhibition assays were carried out in 96-well plates (Nunclon Delta, Thermo Scientific) as described[5, 9]. Briefly, 10^3^ *N*. *crassa* conidia were incubated with 2-fold increasing concentrations of PAF and PAF^D53S/D55S^ (0–128 μM) in liquid medium in a total volume of 200 μL per well. CaCl_2_ was added to the growth medium in a concentration range of 0–5 mM. Caspofungin (Merck) was applied at concentrations of 0.55 mM. Samples were prepared in triplicates. The fungal growth was monitored microscopically and by measuring the optical density (OD_620nm_) after 24–48 h of incubation at 25 °C with a FLUOstar Omega spectrophotometer (BMG Labtech). The growth rates of the treated samples were compared with untreated controls. All experiments were repeated at least twice and statistical calculations were done with Microsoft Excel.

### MD simulation

In order to reveal the dynamical impact of the D53S/D55S aa exchanges, MD simulations were performed both on PAF and on the computationally mutated protein PAF^D53S/D55S^. The PAF structure was derived from the RCSB database[6] (PDB ID: 2MHV) and PAF^D53S/D55S^ was generated *in silico* by removing the Oδ atoms of Asp side chains and renaming the Cγ atoms. The protein models were solvated in octahedral boxes in such a way that the closest distance between the box and the protein was 15 Å and then it was filled up with explicit TIP3P water molecules. The systems were neutralized and the sodium-chloride concentrations were set up to a total 40 mmol/dm^3^. After a short energy minimization, a two-step equilibration was carried out. In the first stage the minimized systems were heated up to 298 K under NVT conditions that lasted 50 ps and followed by a 500 ps long stage under isothermal-isobaric conditions, the protein heavy atoms were kept restrained under the whole equilibration protocol by a force constant of 1000 kJ/mol^-1^nm^-2^. After the equilibration procedure 2 μs long NPT MD simulations were performed using periodic boundary conditions with the aid of virtual sites that allowed the 4 fs step size for integrating the Newtonian laws of motions and all bonds were constrained with the LINCS algorithm. A cutoff of 10.0 Å was used for the Lennard-Jones interactions and short-range electrostatic interactions and the force-switch was applied to smoothly switch the forces between 7.0 Å and 10.0 Å. The long-range electrostatics interactions were calculated by Particel Mesh Ewald summation.

For the coupling of thermal bath, the velocity-rescaling method was used with a coupling constant of 1 ps, while the pressure was regulated with the isotropic Parrinello-Rahman method with 2 ps time constant for coupling. The AMBER99SB-ildn-NMR force field were used throughout the simulations[17–19]. A total of 18 μs all-atom molecular dynamics simulations were carried out to study the details of Ca^2+^ binding ability of PAF and PAF^D53S/D55S^.

The molecular dynamics simulations were performed with the GROMACS 5.1.4.[20], and the trajectories were analysed with the software tools of GROMACS and the Bio3D v2.3 packages[21, 22]. For the visualization of protein structures the UCSF Chimera 1.11.2 software was used[23]. The electrostatic surface maps were calculated with the aid of the DelPhi v6.2 software[24].

### Isothermal titration calorimetry (ITC)

To investigate the Ca^2+^ binding ability of the proteins, MicroCal ITC200 (Malvern Instruments LTD) instrument was used. Thirty-eight microliters of 10 mM CaCl_2_ were titrated in 2 μL steps (90 s delays between the injections) into the measuring cell containing 200 μL of 100 μM protein solution. Proteins and Ca^2+^ were dissolved in the same buffer (10 mM MOPS, 40 mM NaCl, pH = 6.0) in each experiment. All ITC experiments were performed at 25 °C. To determine the heat of dilution, a blind experiment was also set up, where Ca^2+^ was injected to the buffer in the same experimental setup. Furthermore, the control experiments were also run with the same parameters using Na^+^, K^+^ and Mg^2+^ ions, respectively, as ligands. The obtained isotherms were analysed and figures were prepared with Origin 7.0 software (Malvern Instruments LTD).

### NMR measurements, signal assignment and structure calculations

In NMR experiments 1.6 mM protein concentration was applied in phosphate buffer (10 mM Na_3_PO_4_, 40 mM NaCl, 0.04% NaN_3_, pH = 6.0). In case of Ca^2+^ saturation experiments 10 mM MOPS buffer (40 mM NaCl, pH = 6.0) was used instead of phosphate buffer to avoid complex formation. NMR experiments were performed at 298 K using NEO or AVANCE-II type spectrometers (Bruker) at 700 or 500 MHz proton frequency. ^1^H chemical shifts were referenced to DSS (2,2-dimethyl-2-sila-pentane-5-sulfonic acid) and indirect chemical shift referencing was used for ^15^N and ^13^C nuclei calculated from the gyromagnetic ratios. Spectra were processed with TopSpin 3.1 (Bruker) and analysed with CCPNmr Analysis 2.1[25] and CARA 1.8.4[26] software packages, equipped with in house written MATLAB scripts. For sequential resonance assignments 2D ^1^H–^1^H NOESY (130 ms), 3D ^15^N-HSQC-TOCSY (60 ms mixing time) and 3D ^15^N-HSQC-NOESY (130 ms) spectra were used. The completeness of ^1^H- and ^15^N-assignment of PAF-Ca^2+^ was acceptable (86.2 and 94.5%, respectively). However, most unassigned ^1^H atoms were HD or HE hydrogens of lysine (Lys) side chains where high number of NOEs is not expected. For the structure calculation NOE cross peaks were collected from 2D ^1^H-^1^H NOESY and ^15^N-edited 3D HSQC-NOESY spectra. No further structure refinement was used for PAF-Ca^2+^; the structure is based on NOE distance restraints. Disulphide pattern was assumed to be identical with that of the wild-type PAF[27] and was given explicitly as covalent bond restraints during all structure calculations. Cyana 2.1 algorithm was used in combination with ATNOS/CANDID for all structure calculations[28]. As usual, ensembles of 100 structures were calculated and 20 of them with the lowest energy were selected.

For NMR titrations, a series of ^15^N-^1^H HSQC NMR spectra was recorded on 1 mM ^15^N-PAF sample in 10 mM MOPS buffer (40 mM NaCl, pH = 6.0) in order to detect ^15^N-^1^H chemical shift changes in response to the increasing Ca^2+^ concentration of the solution. To avoid dramatic increase of the sample volume and loss of sensitivity, small quantities of high-salt concentration stock solutions were used for the titration (Tables C and D in S1 File). The pH of the solution was checked several times during the titration and found to be stable. Exact positions of the resulting cross-peaks were determined by the parabolic fit function of CCPN[25]. The changing peak positions were followed with the aid of "copy assignment" function of the CCPN package[25]. To obtain equilibrium constants, chemical shift changes were fitted to a single binding model[29, 30] and plotted with an in-house written MATLAB script. ^15^N (70.966 MHz) relaxation data of 0.45 mM/L ^15^N-labelled PAF and of the same solution with 20 equivalent CaCl_2_ for Ca^2+^ bound state (T_1_, T_2_, ^15^N-^1^H NOE) were determined with the standard pulse programs and the Dynamics Center 2.4.3 fitting routine of the manufacturer. Waiting times between scans of 3s (T_1_, T_2_) and 7s (NOE) were allowed. The M2 model of the Lipari-Szabó method[31, 32] was applied, that yielded one global correlation time for the whole protein and the residue specific S^2^ order parameters.

## Results

### Generation of PAF^D53S/D55S^

Pilot MD simulations helped to launch experimental studies on the Ca^2+^ binding of PAF, considering the site-specific mutations at putative binding sites Asp19, Asp53 and Asp55. Since the latter must be the main binding site, the PAF variant PAF^D53S/D55S^ was produced with the *P*. *chrysogenum* based expression system as described recently[14]. Protein yields of 30 mg L^-1^ were reached. The purity and the identity of PAF^D53S/D55S^ were verified by ESI-MS. One peak representing the average molecular mass of 6.187 kDa was detected. The calculated theoretical mass (web.expasy.org/protparam) of the reduced form of PAF^D53S/D55S^ was 6.194 kDa. This suggested that three intra-molecular disulphide bonds were formed in this PAF variant that showed no further post-translational modifications, except for the correct processing of the N-terminus (Figure A in S1 File).

### PAF binds Ca^2+^ ions at Asp53/Asp55 residues

**Fig 1:**
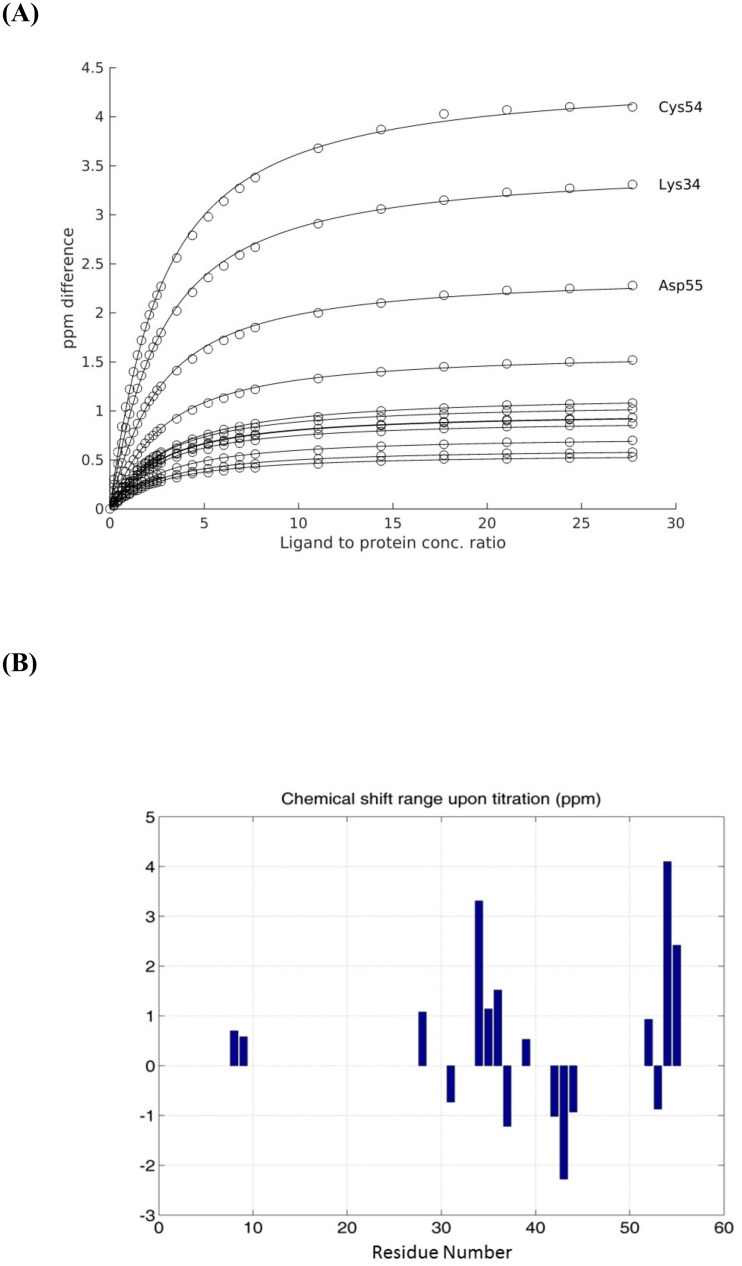
Ca^2+^-sensitive amide nitrogen chemical shift changes of PAF. **(A)** The Ca^2+^-sensitive amide nitrogen chemical shift absolute value changes in PAF as a function of CaCl_2_ concentration as fitted by the two-site fast exchange model. The average equilibrium constant obtained from 12 sites is 463 ± 24 M^-1^. Two traces are nearly overlapping. **(B)** The maximum ^15^N chemical shift changes upon titration as shown against the PAF sequence.

To address the hypothesis that PAF binds Ca^2+^ ions, we performed Ca^2+^ titration experiments using NMR and ITC methods. NMR titrations revealed that the association constant (*K*_a_) averaged for Ca^2+^ sensitive residues of PAF was 463 ± 24 M^-1^ for ^15^N and 490 ± 70 M^-1^ for ^1^H resonances (Fig 1, Figures B and C in S1 File). Furthermore, as expected from its solution structure, it turned out that there is only one major Ca^2+^ binding site in PAF situated at its C-terminus. This observation was also supported by MD simulations as discussed later. Interestingly, the highest ^15^N chemical shift perturbation (> 4 ppm) occurred at cysteine (Cys) 54, in the middle of the predicted and preferred C-terminal binding site. ITC measurements with PAF and Ca^2+^ resulted in similar binding constants as the NMR analysis (*K*_a_ = 400 ± 9 M^-1^) (Figure D in S1 File). Nevertheless, it should be noted that accurate *K*_a_ values are difficult to determine by ITC in low affinity cases (Figure D in S1 File; parameter insets). No effect could be observed in the control experiments, where PAF was missing. To study the specificity of the Ca^2+^ binding, ITC experiments were also performed combining PAF with several other cations, namely Na^+^, K^+^ and Mg^2+^. No interactions were detected in the presence of any of these ions. Next, the Ca^2+^ binding potential of the PAF variant was compared to that of PAF. As expected, the affinity of PAF^D53S/D55S^ to Ca^2+^ was significantly diminished (*K*_a_ = 81 ± 8 M^-1^).

All these findings demonstrate that Ca^2+^ binding to PAF is weak, but specific, and we confirmed unambiguously that Asp53 and Asp55 together with the C-terminal carboxylate group form the preferred Ca^2+^ binding site in PAF.

### Impact of the amino acid substitutions on the antifungal activity

**Fig 2:**
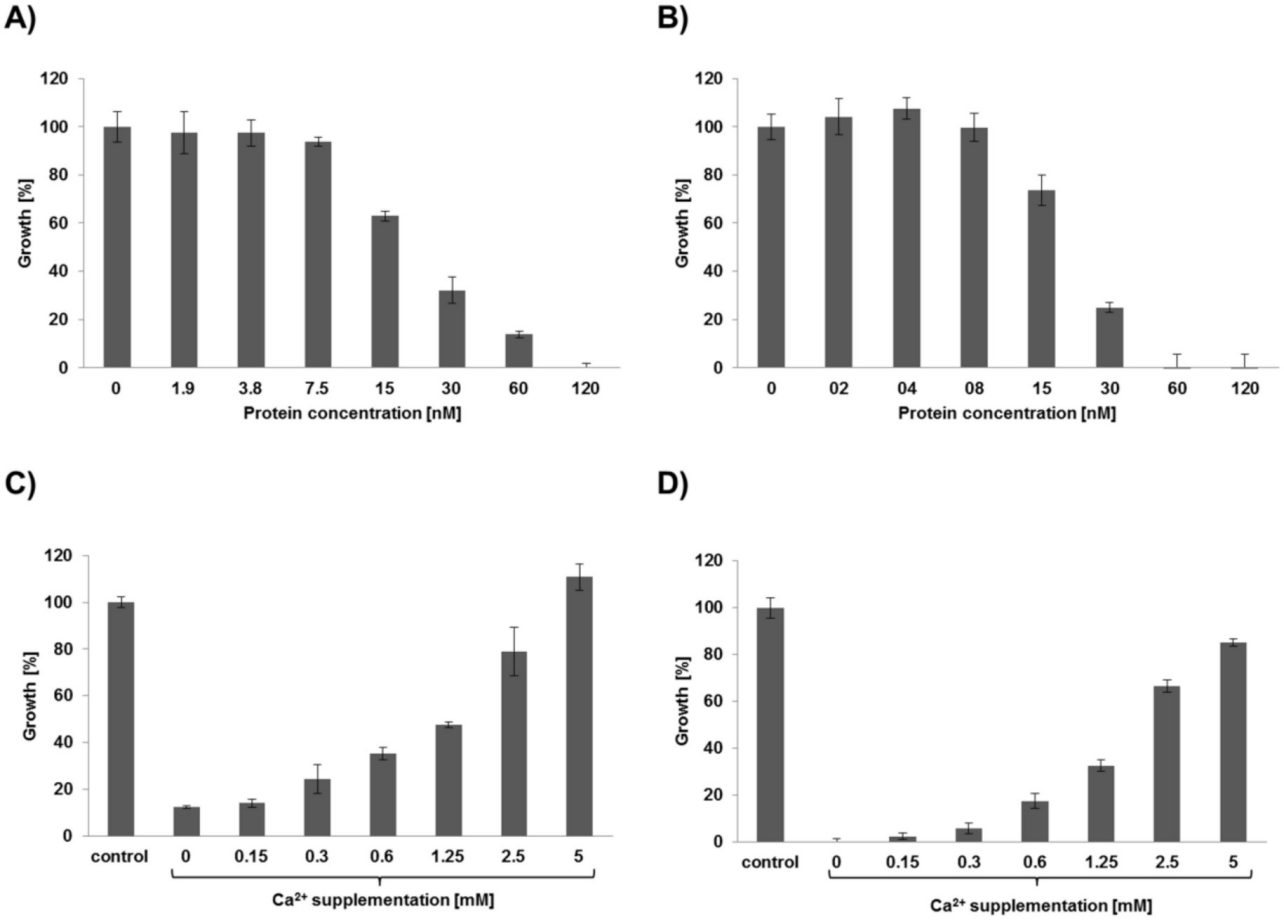
Antifungal activity of PAF^D53S/D55S^ compared to PAF. *N*. *crassa* conidia in 5-fold diluted Vogel´s medium were exposed to increasing concentrations (0–120 nM) of PAF^D53S/D55S^ **(A)** and PAF **(B)** for MEC determination. An effective concentration of 60 nM of PAF^D53S/D55S^ **(C)** and PAF **(D)** was selected to monitor the neutralizing effect of 0–5 mM Ca^2+^ supplementation of the medium on the antifungal efficacy of the proteins. The growth rates were determined after 30 hours of incubation. The untreated controls were set to be 100%. The mean ±SD (technical triplicate of one representative experiment out of two biological replicates) is shown.

The impact of the Asp/Ser substitutions on the antifungal activity was determined by comparing the growth inhibition of PAF^D53S/D55S^ with that of PAF on the test organism *N*. *crassa*. We determined the minimal effective concentration (MEC) that inhibited the growth of *N*. *crassa* germlings by 90%. The MEC for PAF^D53S/D55S^ and PAF was 0.12 μM and 0.06 μM, respectively (Fig 2A and 2B). This indicated that the substitution of Asp53 and Asp55 by Ser did not significantly change the antifungal activity and function of PAF. This result contrasts that of PAF^D19S^, which exhibited significantly reduced antifungal efficacy: the MEC of PAF^D19S^ was 32 μM[8].

As demonstrated earlier, the antifungal activity of PAF and PAF^D19S^ is specifically sensitive to extracellular Ca^2+^ concentrations[8, 9, 33]. To investigate the effect of extracellular Ca^2+^ on the toxicity of PAF^D53S/D55S^, the test medium (0.2 x Vogel’s, containing 0.03 mM CaCl_2_) was supplemented with increasing concentrations of CaCl_2_ (Fig 2C and 2D). The proteins PAF^D53S/D55S^ and PAF effectively inhibited the growth of *N*. *crassa* at a concentration of 0.06 μM. Supplementation of the test medium with CaCl_2_ (0–5 mM) ameliorated the fungal growth in a concentrations dependent way. Five mM CaCl_2_ restored fungal growth to 111 ± 9.6% and 85 ± 2.8% in the presence of PAF^D53S/D55S^ and PAF, respectively. The sensitivity for Ca^2+^ supplementation affecting antifungal efficacy was even higher for the previously described PAF^D19S^[8]. In this mutant, a CaCl_2_ concentration as low as 0.3 mM, restored fungal growth to 50%[8]. Fungal growth recovery was significantly lower at this CaCl_2_ concentration and reached only 12% in PAF^D53S/D55S^ treated and 6% in PAF treated samples.

To verify the specificity of our results, we conducted experiments in which the proteins were replaced with caspofungin, which inhibits the β-1,3 glucan synthase and perturbs the cell wall synthesis of metabolically active fungi[34], thus exhibiting a different mechanistic antifungal function. We could not detect any influence of supplementing the growth medium with CaCl_2_ on the antifungal activity of caspofungin against *N*. *crassa* (Figure E in S1 File). This further corroborated our finding that the Ca^2+^ sensitivity is specific for the mode of action of PAF and its variants[8].

### PAF, PAF-Ca^2+^ and PAF^D53S/D55S^ exhibit similar solution structures

**Fig 3:**
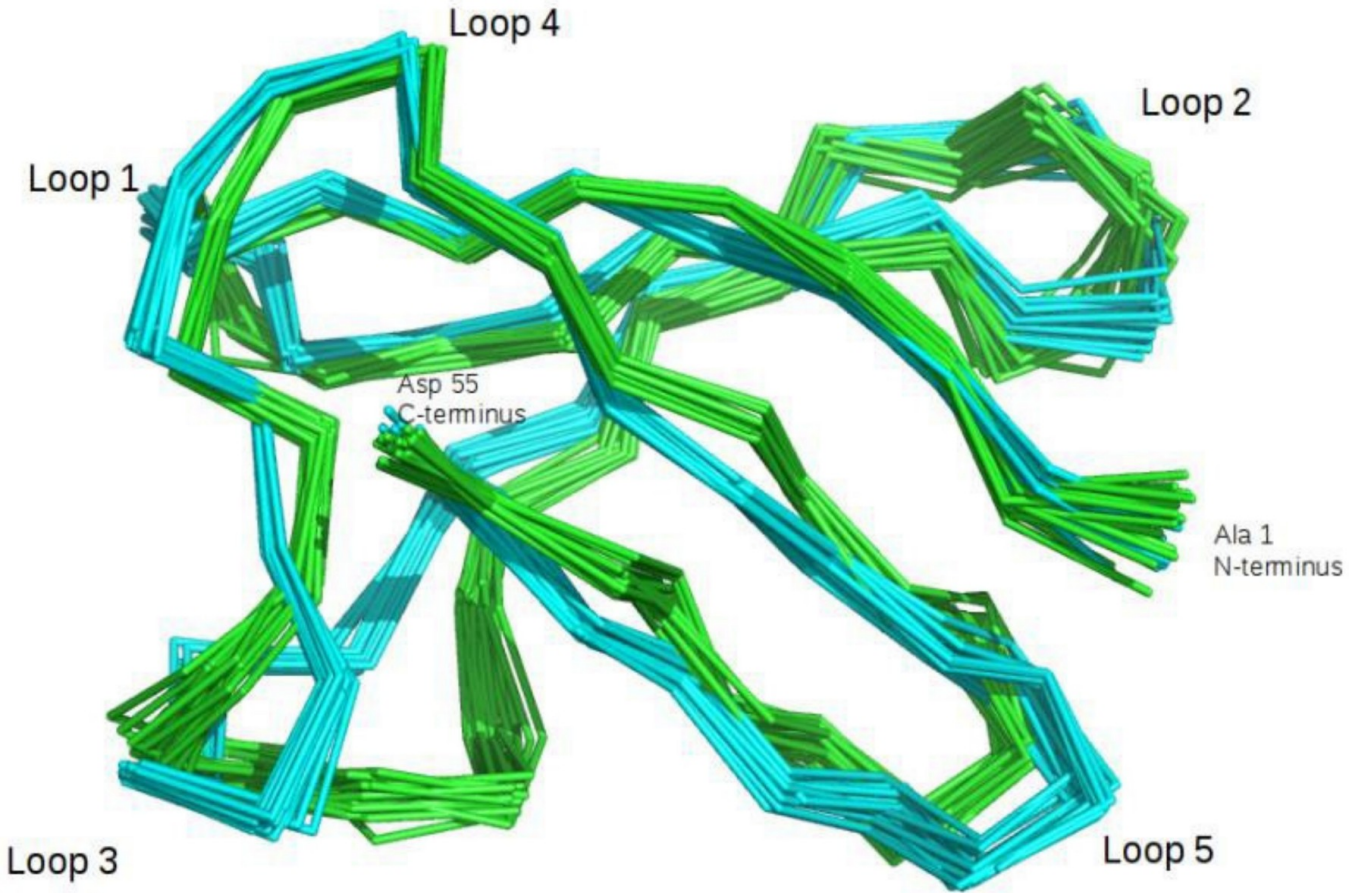
Alignment of PAF structure (cyan, PDB ID: 2MHV) with the structure of PAF-Ca^2+^ (green, PDB ID: 2nbf). Protein backbone is represented as ribbons of the best 20 conformers during structure calculation.

Since the titration experiments by NMR and ITC methods revealed a significant difference in Ca^2+^ binding affinity between PAF and PAF^D53S/D55S^, we concentrated our further studies on these two protein variants. Our first goal was to investigate potential structural changes of PAF[6] caused by Ca^2+^ binding. Thus, the three-dimensional solution structure of PAF-Ca^2+^ was determined (PDB ID: 2NBF, BMRB ID: 25974). In the ^1^H-^15^N HSQC spectra of PAF-Ca^2+^ all amide NH-s and Asp side chain NH_2_ resonances were well dispersed indicating the folded state of PAF-Ca^2+^. With ^15^N-labelled PAF the average number of NOE distance restraints in the complex was 14.7 per residues, which is slightly lower than for the ^15^N-/^13^C-labelled PAF structure[6]. Consequently, the 3D structure of PAF-Ca^2+^ is less well defined, especially at the N-terminus and in some loops regions. However, the quality of the Ca^2+^-bound structure was sufficient for comparison with the Ca^2+^-free PAF (Figure F in S1 File and Fig 3). The secondary structure analysis clearly indicated that all five β-strands were also present in the Ca^2+^-bound state. In general, the PAF-Ca^2+^ has the same global fold as PAF showing two antiparallel β-pleated sheets, four loop regions and the hidden central core formed by three disulphide bonds[5]. The conformation of loop 3 (Lys30-Asp39) showed a slight variation in PAF-Ca^2+^ (Figure F in S1 File and Fig 3). Thus, the first β-turn (Asp32-Asn33) present in PAF can only be defined as a turn-like conformation here. Still, the large loop 3 adopts a bend with β-turn at Asp39-Asn40, like in the Ca^2+^-free state, suggesting that its structure remained fairly similar in PAF-Ca^2+^ and PAF. The large loop 3 in the PAF-Ca^2+^ is less well defined due to the lower number of NOEs, that could also explain the small differences between the holo and apo forms. In the PAF-Ca^2+^ the second β-pleated sheet proved to be less well organized. Moreover, the C-terminal β-strand was slightly irregular and much shorter (Ala51-Asp53). This conformational change increased the negative charge of the protein surface at the C-terminus, which supported the theoretical calculations indicating Asp53 and Asp55 to be the key residues for Ca^2+^ binding in this region (Figure F in S1 File). However, the negatively charged C-terminus lacks a deep binding pocket, which might explain the weak Ca^2+^ binding affinity of PAF.

Next, we determined the effect of substituting the aa Asp53 and Asp55 with Ser on the protein solution structure. Therefore, PAF^D53S/D55S^ was ^15^N-labelled and analysed by NMR spectroscopy (BMRB ID: 26734). The ^15^N-^1^H chemical shift dispersion pointed towards a similarly folded structure as that of PAF. This was further supported by the well comparable ^13^C-chemical shifts from natural abundance ^13^C-HSQC spectra, which indicated similar backbone conformation (Table E in S1 File, Figures G and H in S1 File). Thus, we conclude that the 3D structure of PAF^D53S/D55S^ is essentially the same as that of PAF.

### MD simulations of PAF, PAF-Ca^2+^ and PAF^D53S/D55S^

Four sets of simulations were run to study the Ca^2+^ binding ability of PAF and PAF^D53S/D55S^ (Table F in S1 File): (1) In the first set the native and the computationally generated PAF^D53S/D55S^ were examined under biologically relevant conditions (40 mM NaCl) in the absence of Ca^2+^ ions. (2) In the second set the same simulation systems were built up in the presence of one Ca^2+^ ion which means approximately 7 mM (corresponding to the whole volume of the simulation box). (3) To study the protein dynamics and the effect of high Ca^2+^ concentration, in the third set ten Ca^2+^ ions were placed into the simulation box, which fit to a super-saturated simulation cell and an approx. 70 mM calcium ion concentration. (4) The last three runs were controls to prove that the MD simulations are able to clearly distinguish the selective ion binding ability of proteins. In these simulations, we placed one Mg^2+^ ion, then one Mg^2+^ and one Ca^2+^ and finally two Ca^2+^ ions into the simulation box.

**Fig 4:**
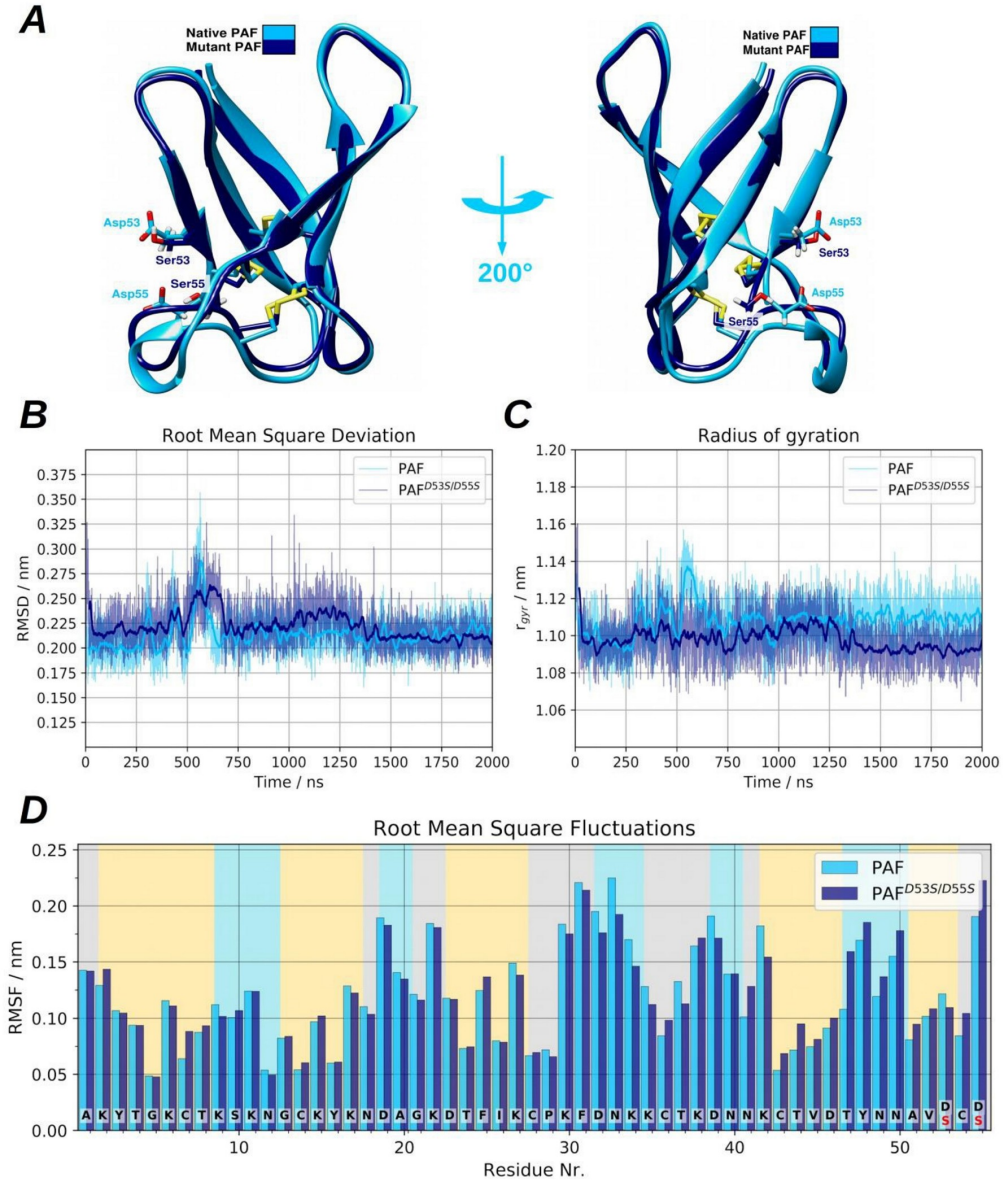
Graphical representation of PAF and PAF^D53S/D55S^ and side-by-side comparison of protein dynamics. **(A)** The aligned native and mutant PAF structures can be seen after clustering the MD trajectories and displaying the representatives of most populated clusters. **(B)** The heavy atom RMSD and **(C)** the radius of gyration are depicted. **(D)**The per-residue root mean square fluctuation (RMSF) can be seen, calculated to all heavy atoms.

(1) Panel A in Fig 4 shows that the position of the Asp53 to Ser and Asp55 to Ser mutations located at the end of the β-strand 5 and the strand itself is bound to the end of β-strand 3 via a disulphide bridge between Cys54 and Cys28. After analysing the MD trajectories, the representatives of the most populated clusters showed that the average backbone root mean square deviations (RMSD) between PAF and PAF^D53S/D55S^ was only 0.46 Å. The RMSD revealed no significant discrepancies, nevertheless after the first 1.25 μs of the simulation, the total radius of gyration suggested a somewhat more compact structure in the case of PAF^D53S/D55S^, as can be seen in panel B and C of Fig 4, respectively. Conspicuous differences can be seen in the region of phenylalanine (Phe)31-Lys42 (loop 3) and Cys43-Ser55 (β-strands 4 and 5). Although the long loop region indicates a slightly higher fluctuation, the last two sheets in PAF^D53S/D55S^ became more rigid compared to those in PAF. The reason for these observations is related to the change of salt bridge patterns on the protein surface (Figure P in S1 File). Furthermore, the per-residue root mean square fluctuations (RMSF) indicated deviations mostly in the above-mentioned regions (Fig 4D). The alteration of Lys-Asp salt bridge connections reflected the dynamics of torsion angles around the disulphide bonds and *vice versa* (Figure I in S1 File). The reason for these observations is that each Cys has a Lys residue in proximal position, except for Cys54 which is embedded between two Asp and hence it can perceive changes in the salt-bridge patterns. While PAF is able to create typical salt bridge connections via Asp53, Asp55 and the C-terminus, the corresponding β-strands turn to be somewhat more rigid (as the RMSF data suggests). In the case of PAF^D53S/D55S^ the C-terminal Ser53 and Ser55 are obviously unable to evolve salt-bridge connections, constrained the Lys side chains to adopt new, favourable conformations, hence causing higher C-terminal fluctuations. Based on the cluster representatives, the computed electrostatic surface maps show major differences between PAF and PAF^D53S/D55S^, as expected. While PAF has a negatively charged region near the Asp53 and Asp55 suitable to bind a Ca^2+^ ion, the region of Ser53 and Ser55 is more positively charged in PAF^D53S/D55S^ (Figure J in S1 File). Apart from these minor changes, the PAF^D53S/D55S^ structure is similar to PAF, and the aa substitutions did not cause significant structural deviations or unfolding events on the simulated 2 μs long time scale.

The most significant changes of ^1^H-^15^N chemical shifts upon Ca^2+^ titration were observed near to the Ca^2+^ binding site (Figure K in S1 File) suggesting possible structural differences between the Ca^2+^-free PAF and PAF-Ca^2+^. Taking the representatives of the most populated clusters, the secondary structure analysis revealed only minor alterations in the backbone conformation with an average 0.40 Å RMSD. Hence both structures were very similar to each other (Figure K in S1 File). The absolute difference between the generalized correlation matrices[35] of backbone atoms (Panel B of Figure K in S1 File) is in agreement with the changes of chemical shifts. This reveals that the most significant changes occur in those regions where the chemical shifts also alter (Lys34-Thr37, Lys42-Thr44, Val52-Asp55). The trend of the calculated S^2^ values from MD trajectories were in line with the experimental ones, though the S^2^ from MD is obtained at longer (μs vs. ns) timescales (Panel A of Figure L in S1 File).

**Fig 5:**
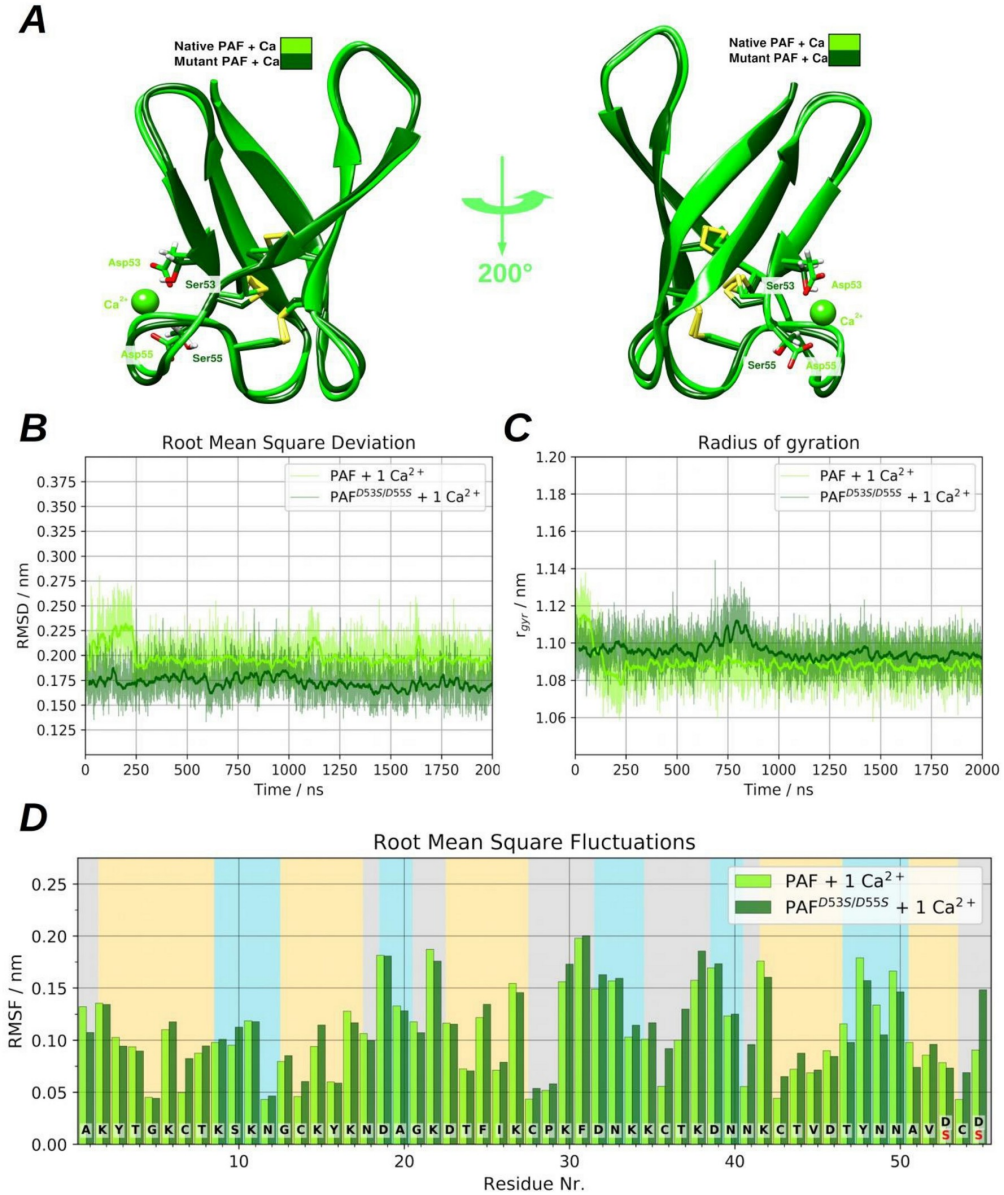
Graphical representation of the PAF and PAF^D53S/D55S^, side-by-side comparison of protein dynamics in the presence of one Ca5^2+^ ion. **(A)** Aligned PAF and PAF^D53S/D55S^ structures can be seen after clustering the MD trajectories and selecting the representatives of most populated clusters. **(B)** shows the heavy atom RMSD. **(C)** depicts the radius of gyration. **(D)** per-residue root mean square fluctuation (RMSF) can be seen and calculated to all heavy atoms.

**Fig 6:**
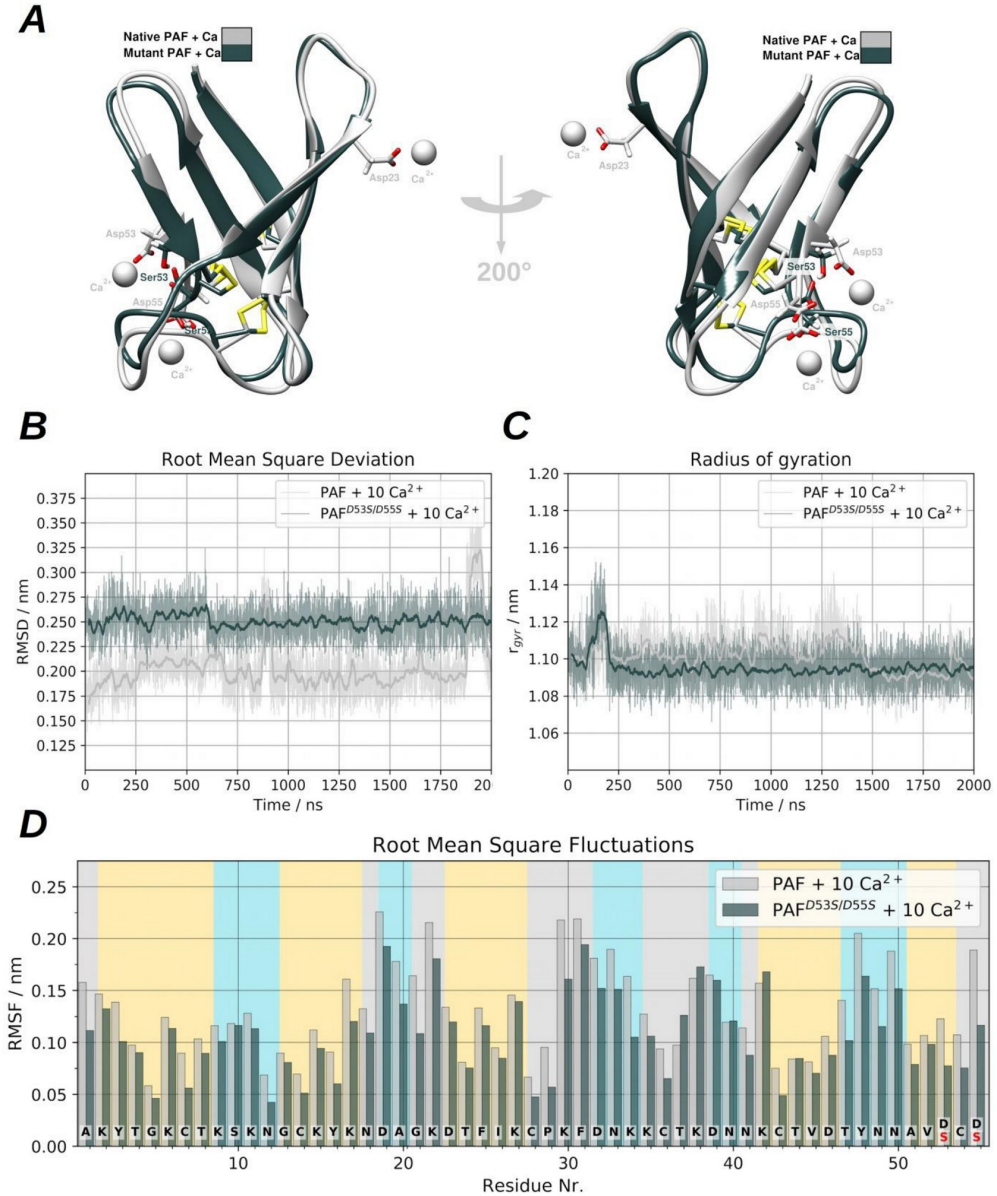
Graphical representation of the PAF and PAF^D53S/D55S^, side-by-side comparison of protein dynamics in the presence of ten Ca^2+^ ions. **(A)** Alignment of PAF and PAF^D53S/D55S^ structures can be seen after clustering the MD trajectories and showing the representatives of most populated clusters. **(B)** shows the heavy atom RMSD, **(C)** depicts the radius of gyration. **(D)** the per-residue root mean square fluctuation (RMSF) can be seen and calculated to all heavy atoms.

(2) The diminished Ca^2+^ binding ability of PAF^D53S/D55S^ is obviously related to the lack of negatively charged side chains of Asp53 and Asp55. Indeed, simulations in the presence of one Ca^2+^ ion clearly demonstrated that PAF has an ability to bind Ca^2+^ at its C-terminal end by these residues and the C-terminal carboxylate group (Fig 5A). As opposed to the triple claw-like Ca^2+^ binding site of PAF, PAF^D53S/D55S^ is unable to bind Ca^2+^at its C-terminus (Panel C of Figure M in S1 File); however, the Asp32 could form a weak binding site. Both, PAF and PAF^D53S/D55S^ show similar dynamical properties, which were observed in the absence of Ca^2+^ (Fig 5B–5D). Although small differences can be noticed in the RMSD and in the per-residue fluctuations, the radiuses of gyrations are almost identical. PAF shows somewhat larger RMSD than PAF^D53S/D55S^ which is clearly connected to Ca^2+^ binding. In the first 250 ns, the C-terminal negatively charged groups adopt a new conformation and in the remaining part of simulation the Ca^2+^ binding keeps the β-strand 5 rigid and the RMSF values also confirm this observation while the radiuses of gyrations are pretty close to each other. Compared to the simulations without Ca^2+^, in the case of PAF^D53S/D55S^ we can see almost the same gyration radius (~1.09 nm) but in the case of PAF the radius of gyration is significantly decreased (from ~1.11 nm to ~1.09 nm), confirming again that the Ca^2+^ binding results a less flexible, compact structure.

(3) Due to the fact that some NMR measurements were performed in Ca^2+^ rich media, several simulations were conducted under similar condition, which means approx. 70 mM Ca^2+^ above the existing 40 mM NaCl. We could find differences between the used general dynamical descriptors of PAF and PAF^D53S/D55S^ (Fig 6) and the salt bridge distances and also in the Ca^2+^ binding properties. The Figure N in S1 File shows that several other temporary binding sites exist under these conditions, both in PAF and PAF^D53S/D55S^. It should be noted that changes observable in the Lys-Asp salt bridge distances and their formation are obviously related to the artificially high Ca^2+^ concentration that leads to the formation of weak binding modes. In the case of the hereby studied proteins these weak or temporary sites are very similar. This suggests identical dynamical behaviour of PAF and PAF^D53S/D55S^. Ca^2+^ induced salt bridge formation however, might be important in the case of Ca^2+^ regulated enzymes. The logic behind these observations are (a) the unusually high ion strength can induce temporary Ca^2+^ binding sites (Figure N in S1 File); (b) the formation of temporary sites is clearly in connection with the salt bridge distances on the protein surface (Panel B of Figure O and Panel A of Figure P in S1 File); (c) changes in salt-bridge connections where at least the donor or acceptor residue has an adjacent Cys, can be mediated to the protein core via the disulphide bridges. Statements (b) and (c) are generally true even if there are no Ca^2+^ ions in the corresponding system, for example in our first simulation set (Panels A and B of Figure I in S1 File).

(4) We could show that PAF has a weak Ca^2+^ binding site but does not interact with other cations such as K^+^, Na^+^ or Mg^2+^. To verify that our MD investigations are able to correctly predict Ca^2+^ (and other cation) binding properties of PAF, three additional simulations were performed. In the first simulation cell one Mg^2+^ ion was placed randomly and retained its position, one Ca^2+^ was added, and finally two Ca^2+^ ions were considered in the simulation box. The general analysis of these simulations can be found in Figures Q and R in S1 File. Based on these systems we conclude that the Mg^2+^ accessed the negative side-chains only temporarily as well as in the Ca^2+^-Mg^2+^ containing box, Ca^2+^ can be found in the C-terminal binding pocket (Figure S in S1 File). In the case of two Ca^2+^ ions, one of them was located in the C-terminal pocket and the other one circling around the protein freely, taking a ~50 ns long stop at the Asp19 at the end of the simulation (Panel C of Figure S in S1 File).

## Discussion

In this study, we investigated the hypothesis that binding of extracellular Ca^2+^ to PAF might be the reason for the loss of its antifungal activity. Our investigations shed new light on the Ca^2+^ binding ability outside the fungal cell and its impact on the structure-function relation of the antifungal protein PAF, which may support our understanding of the mode of action of other PAF- related antifungal proteins from filamentous fungi.

We proved by NMR spectroscopy and MD simulations that PAF primarily binds Ca^2+^ at its C-terminus (Asp53 and Asp55). The binding is rather weak (K_a_ ~ 460/M), which may be explained by the absence of a well-formed binding pocket in PAF. No interactions between PAF and other cations (Na^+^, K^+^ and Mg^2+^) could be detected. This confirms selective Ca^2+^ binding of PAF, though we wondered why this interaction is weak. Therefore, the Asp mutants PAF^D53S/D55S^ and the previously described PAF^D19S^[8] were carefully examined and compared to the wild-type protein. As expected, Ca^2+^ ions showed significantly reduced binding to PAF^D53S/D55S^. Since the secondary Ca^2+^ binding site at Asp19 is less probable, its mutation in PAF^D19S^ has little impact on the overall Ca^2+^ binding affinity. Characteristic ^13^C NMR chemical shifts suggested that the exchange of two negatively charged Asp at the binding site in PAF^D53S/D55S^ did not affect appreciably the overall solution structure with respect to PAF. The same structural stability is true for PAF^D19S^ as discussed earlier[8]. In the PAF-Ca^2+^ complex structure we observed small differences in the big loop 3, whereas the second β-pleated sheet has become slightly shorter and less regular compared to PAF. Still, the global fold and the 3D structure is almost the same both in the apo and holo forms, i.e. Ca^2+^ binding to PAF (PDB ID: 2NBF for bound state) does not influence its essential structure. The MD of PAF and PAF^D53S/D55S^ were extensively studied by all-atom MD simulations, including the Ca^2+^ binding features. We could prove that PAF^D53S/D55S^ cannot bind Ca^2+^ ions; a consequence of missing Asp53 and Asp55 side chains, that causes the change of long-range electrostatic surface, as the only significant difference we found between PAF and PAF^D53S/D55S^.

Experimental (2NBF) and calculated (MD simulations, Figure T in S1 File) structures of PAF-Ca^2+^ were in acceptable agreement (RMSD = 2.19 Å). MD simulations gave new insights into the dynamics of disulphide bonds and salt bridges in PAF and PAF^D53S/D55S^ in accordance with NMR chemical shift movements upon Ca^2+^ titration of PAF. The overall PAF dynamics represented by the average ^15^NH S^2^ order parameters did change upon Ca^2+^ binding neither by experimental (0.83 ± 0.05 vs. 0.83 ± 0.07) nor theoretical (0.84 ± 0.06 vs. 0.83 ± 0.07) results. Locally however, the experimental S^2^ order parameter differences (obtained from ^15^N NMR relaxation)[31, 32] displayed reduced dynamics in the protein region between aa 34–37 (Panel B of Figure L in S1 File). For these phenomena, a possible explanation could be the spatial proximity of the β-strands 4 and 5, suggesting an interference between the salt and disulphide bridges. In other words, changes on the protein surface are mediated to the hydrophobic core, moreover the core where the cysteines can be found, can also distribute information not only via directly linked disulphide bonds, but between other cysteines as well (Cys54 is not exclusively correlated with Cys28; Cys43, Cys36, Cys14, Cys7 and so on, see arrows in Panel B of Figure K in S1 File.

It also turned out that the antifungal activity of PAF^D53S/D55S^ tested on *N*. *crassa* is very similar to that of PAF (MEC values of 0.13 and 0.06 μM, respectively). The antifungal activity of the recently studied protein variant PAF^D19S^ was highly sensitive[8] to Ca^2+^ ions, while PAF^D53S/D55S^ exhibited a similar Ca^2+^ ion dependent antifungal activity as PAF. Hence, similar Ca^2+^ -dependent activities may be explained by the structural and dynamical similarities of PAF and PAF^D53S/D55S^. In summary, we conclude that the Ca^2+^ sensitive mode of the PAF action is independent of its direct Ca^2+^ binding capability, corroborating the earlier hypothesis that extracellular Ca^2+^ ions regulate the fungicidal mode of action of PAF indirectly in a more complex way, e.g. by influencing the expression and/or activity of PAF-specific interaction molecules/receptors or Ca^2+^ transporters in fungal cells[8, 9].

## Supporting information

S1 File **Supplementary Figures and Tables: Table A. Fungal strains used in this study. Table B. Oligonucleotides used in this study**. Mutation primers are in bold; mismatches for aa exchange are underlined. **Table C. Setup of NMR titration of native PAF with CaCl**_**2**_. **Table D**. ^**15**^**N Chemical shift changes (ppm) of the Ca**^**2+**^**-sensitive residues of PAF in the function of CaCl**_**2**_ **concentration. Table E. C**_**α**_ **and C**_**β**_ **chemical shifts (ppm) of PAF and PAF**^**D53S/D55S**^. Missing resonances are due to low intensity NH and NH correlated peaks which is a consequence of H/D exchange of these peaks at pH = 6.0. **Table F. Ion binding probabilities of PAF and PAF**^**D53S/D55S**^ **proteins**. Calculations of ion binding probabilities were based on the number of frames where the ion was closer to the corresponding carboxylate carbon than 0.4 nm, divided by the total number of frames multiplied by 100. Only those values are shown which differed from 0.00%. **Figure A. Molecular mass determination of PAF**^**D53S/D55S**^ **by ESI-MS. (A)** Overview on the average isotopic pattern spanning 4–8 kDa identifies the correct mass of PAF^D53S/D55S^ (6.187 kDa). **(B)** Detailed view (6.1–6.6 kDa) reveals that additional peaks can be attributed to chemical adducts. MS results for PAF and PAF^D19S^ were published previously [5,8]. **Figure B. *K***_**e**_ **value of Ca**^**2+**^ **binding of PAF calculated for individual aa residues**. Ca^2+^ binding constants as determined from the fit of ^15^N chemical shifts upon Ca^2+^ titration yielded similar results, independent of the sequential distance from the effective binding site. **Figure C. Ca**^**2+**^**-sensitive amide proton chemical shift changes of PAF (MOPS buffer) upon CaCl**_**2**_ **titration**. Experimental points were fitted as a function of CaCl_2_ concentration according to the one site binding model. Equation 3 was used from reference [30] for fitting the absolute value of ^1^H chemical shift changes by using an in-house written MATLAB code. The average equilibrium constant *K*_a_ = 490 ± 70 M^-1^ is in agreement with ^15^N and ITC results. **Figure D. ITC isotherms for CaCl**_**2**_**-titrations. (A)** PAF **(B)** MOPS buffer (negative control) **(C)** PAF^D19S^ **(D)** PAF^D53S/D55S^. **Figure E**. Antifungal effect of Caspofungin (1.25 μg/mL) on *Neurospora crassa* is not affected by increasing concentrations of extracellular CaCl_2_ (0–10 mM). **Figure F**. Top: Cartoon model of Ca^2+^-bound PAF structure (left, green, PDB ID: 2nbf) and structure of Ca^2+^-free PAF (right, cyan, PDB ID: 2mhv). Beta-strands are shown as flat arrows, and disulphide-bridges are represented in yellow. Bottom: Surface representation of Ca^2+^-bound PAF (left) and Ca^2+^-free PAF (right) coloured according to electrostatic potential calculated in vacuum (blue: electropositive; red: electronegative). A negatively charged patch can be seen at the C-terminus of the protein. **Figure G. C**_**α**_ **chemical shifts of PAF, PAF**^**D19S**^ **and PAF**^**D53S/D55S**^. **Figure H. C**_**β**_ **chemical shifts of PAF, PAF**^**D19S**^ **and PAF**^**D53S/D55S**^. **Figure I. Comparison of the dynamics of disulphide dihedrals and salt bridge distances in the absence of Ca**^**2+**^ **ion. (A)** Changes of the Cβ-Sγ-Sγ-Cβ torsion angle (native and mutant respectively). **(B)** The C-terminal related salt bridge distances are shown and correspond to the distances between the Lys Nζ—Asp Cδ, Lys Nζ—Ser Oγ, and Lys Nζ—C-terminal carboxylate carbon atoms. **Figure J. Electrostatic potential surfaces of PAF and PAF**^**D53S/D55S**^ **mutant proteins. (A)** In the case of PAF, the electrostatic surface map clearly shows a negative surface potential in the vicinity of Asp53 and Asp55, which explains the Ca^2+^ binding ability of the native protein. **(B)** In contrast to PAF, the PAF^D53S/D55S^ shows significantly positive surface at the same position (ESP values shown in kT units). **Figure K. Secondary structures of cluster representatives of PAF (without Ca**^**2+**^**/with 10 Ca**^**2+**^**, respectively) and differences in the generalized correlations and in the chemical shifts. (A)** Graphical representations of secondary structure elements (cartoon) of most populated cluster representatives of PAF in the absence of Ca^2+^ ions (left), and in the presence of ten Ca^2+^ ions (right), residues with notable chemical shift change were depicted with purple (ball and stick). In panel B above, absolute difference between generalized covariance matrices, on the edges the individual secondary structure elements were depicted (vertical axis: absence of calcium, horizontal axis: presence of 10 Ca^2+^). The absolute difference of matrices was calculated by: |C_ij_^PAF^ − C_ij_^PAF-10Ca2+^|. **(B)** Chemical shift differences upon titration experiments, the background color represents the SSEs of the saturated PAF. **Figure L. Theoretical and experimental *S***^**2**^ **order parameters. (A)** Comparison of NMR experimental (pink) and MD theoretical (cyan) *S*^2^ order parameters of free PAF. Missing point is Pro29 due to the lack of an NH group. The overall trends of theoretical and experimental *S*^2^ are similar, and differences might be attributed to the different time scopes (μs vs. ns) of the methods and the experimental uncertainties. **(B)** NMR experimental differences between Ca^2+^ loaded and free PAF. Error bars (yellow) are shown on the top of the differences (blue). Positive bars mean rigidification upon binding, while negative differences mean enhanced dynamics. **Figure M. Comparison of the dynamics of disulphide bonds and salt bridge distances in the presence of one Ca**^**2+**^ **ion. (A)** Changes of the Cβ-Sγ-Sγ-Cβ torsion angle (native and mutant respectively). (**B**) The C-terminal related salt bridge distances are shown and correspond to the distances between the Lys Nζ—Asp Cδ, Lys Nζ—Ser Oγ, and Lys Nζ—C-terminal carboxylate carbon atoms. **Figure N. Minimum distances between Ca**^**2+**^ **ions and negatively charged side-chains**. Those residues were considered where the Ca^2+^ binding was clearly observable. In the left panel PAF is depicted, in the right panel the PAF^D53S/D55S^ mutant. These simultaneous, artificial binding sites can be induced by the high ionic strength but clearly demonstrate that the most effective binding can occur at the C-terminus of PAF. **Figure O. Comparison of the dynamics of disulphide bonds and salt bridge distances in the presence of ten Ca**^**2+**^ **ions. (A)** Changes of the Cβ-Sγ-Sγ-Cβ torsion angle (native and mutant respectively). **(B)** The C-terminal related salt bridge distances are shown and correspond to the distances between the Lys Nζ—Asp Cδ, Lys Nζ—Ser Oγ, and Lys Nζ—C-terminal carboxylate carbon atoms. **Figure P. Salt bridge distances in PAF and PAF**^**D53S/D55S**^. The Ca^2+^ concentration induced changes are shown in **(A)** for PAF, in **(B)** for PAF^D53S/D55S^, depicted and based on the distance between the Lys Nζ—Asp Cδ, Lys Nζ—Ser Oγ, and Lys Nζ—C-terminal carboxylate carbon atoms. **Figure Q. Salt bridge distances in control simulations**. The Ca^2+^ and Mg^2+^ concentration induced changes in the salt bridge connections are shown for PAF **(A)** one Mg^2+^, **(B)** one Mg^2+^ and one Ca^2+^, **(C)** two Ca^2+^, based on the distances between the Lys Nζ—Asp Cδ, Lys Nζ—Ser Oγ, and Lys Nζ—C- terminal carboxylate carbon atoms. **Figure R. General dynamical properties of control simulations. (A)** and **(B)** The MD simulations are summarized and based on calculations to all heavy atoms, in **(C)** the C-terminal related salt-bridge distances are collected and correspond to the distances between Lys Nζ—Asp Cδ or C-terminal carboxylate carbon atoms. **Figure S. Minimum distances of** Ca^2+^ **and** Mg^2+^ **ions in control simulations. (A)** The applied Mg^2+^ concentration does not result in any significant binding. **(B)** and **(C)** In the case of one or two Ca^2+^, the C-terminal binding site was filled in the second half of the simulations and in the latter case the second Ca^2+^ ion bound weakly to the Asp19 in the last ~50 ns. The corresponding values fit to the distances between the ion and the Asp Cδ or Ser Oγ or C-terminal carboxylate carbon atoms. **Figure T. Comparison of the experimental (2NBF, red) and theoretical (MD simulation, blue) structures of Ca**^**2+**^ **loaded PAF**. The RMSD between the representative of the largest cluster and the first frame of 2NBF is 2.19 Å.
(DOCX)

S2 File NMR structure validation report of PAF-Ca^2+^ (PDB ID: 2NBF). (PDF)
