# Supplementary material for: Calcium binding of the antifungal protein PAF: Structure, dynamics and function aspects by NMR and MD simulations
Source: PLoS One. 2018 Oct 15;13(10):e0204825. doi: 10.1371/journal.pone.0204825 (PMC6188699; doi:10.1371/journal.pone.0204825)
Supplement: S1 File — Supplementary Figures and Tables: Table A. Fungal strains used in this study. Table B. Oligonucleotides used in this study. Mutation primers are in bold; mismatches for aa exchange are underlined. Table C. Setup of NMR titration of native PAF with CaCl2. Table D. 15N Chemical shift changes (ppm) of the Ca2+-sensitive residues of PAF in the function of CaCl2 concentration. Table E. Cα and Cβ chemical shifts (ppm) of PAF and PAFD53S/D55S. Missing resonances are due to low intensity NH and NH correlated peaks which is a consequence of H/D exchange of these peaks at pH = 6.0. Table F. Ion binding probabilities of PAF and PAFD53S/D55S proteins. Calculations of ion binding probabilities were based on the number of frames where the ion was closer to the corresponding carboxylate carbon than 0.4 nm, divided by the total number of frames multiplied by 100. Only those values are shown which differed from 0.00%. Figure A. Molecular mass determination of PAFD53S/D55S by ESI-MS. (A) Overview on the average isotopic pattern spanning 4–8 kDa identifies the correct mass of PAFD53S/D55S (6.187 kDa). (B) Detailed view (6.1–6.6 kDa) reveals that additional peaks can be attributed to chemical adducts. MS results for PAF and PAFD19S were published previously [5,8]. Figure B. Ke value of Ca2+ binding of PAF calculated for individual aa residues. Ca2+ binding constants as determined from the fit of 15N chemical shifts upon Ca2+ titration yielded similar results, independent of the sequential distance from the effective binding site. Figure C. Ca2+-sensitive amide proton chemical shift changes of PAF (MOPS buffer) upon CaCl2 titration. Experimental points were fitted as a function of CaCl2 concentration according to the one site binding model. Equation 3 was used from reference [30] for fitting the absolute value of 1H chemical shift changes by using an in-house written MATLAB code. The average equilibrium constant Ka = 490 ± 70 M-1 is in agreement with 15N and ITC results. Figure D. ITC i [file pone.0204825.s001.docx]

**Supporting information files**

**Calcium binding of the antifungal protein PAF: structure, dynamics and function aspects by NMR and MD calculations**

# Ádám Fizil1, Christoph Sonderegger2, András Czajlik1, Attila Fekete1, István Komáromi3, Dorottya Hajdu1, Florentine Marx2* and Gyula Batta1*

**1**Department of Organic Chemistry, Faculty of Science and Technology, University of Debrecen, Debrecen, Hungary

**2**Division of Molecular Biology, Biocenter, Medical University of Innsbruck, Innsbruck, Austria

**3**Division of Clinical Laboratory Science, Department of Laboratory Medicine, Faculty of Medicine, University of Debrecen, Debrecen, Hungary

## *Corresponding Authors

E-mail: [batta@unideb.hu](mailto:batta@unideb.hu) (GB),

[florentine.marx@i-med.ac.at](mailto:florentine.marx@i-med.ac.at) (FM)

**Tables**

**Table A**

| **Strain** | **Genotype** | **Reference** |
| --- | --- | --- |
| *N. crassa* 74-OR23-1A | wild-type mat A | FGSC 2489 |
| *P. chrysogenum* *∆paf* | *∆paf*:*nat1* | [16] |
| *P. chrysogenum paf* | *Δpaf*:*nat1, paf*, *ptrA*^+^ | [14] |
| *P. chrysogenum paf*^D19S^ | *Δpaf*:*nat1, paf*^D19S^, *ptrA*^+^ | [8] |
| *P. chrysogenum paf*^D53S/D55S^ | *Δpaf*:*nat1, paf*^D53S/D55S^, *ptrA*^+^ | this study |

**Table B**

| **Name** | **Sequence 5'-3'** |
| --- | --- |
| M13 | GTAAAACGACGGCCAGTGAG |
| T7var | TACGACTCACTATAGGGCG |
| opaf11 | CACTCCCCTCATACTTCATG |
| opaf12 | CTTCTCTGACTGAAAGTACC |
| **opafD53S/D55Sfw** | **GTCTCCTGTTCCTAGATGGTC** |
| **opafD53S/D55Srev** | **GACCATCTAGGAACAGGAGAC** |

**Table C**

| **Injection number** | ***V*_CaCl2_ added [µL]** | ***V* sample [**µ**L]** | ***C*_CaCl2_ added [mM]** | ***C*_Protein_ [mM]** | ***C*_CaCl2_ [mM]** | ***n*_PAF_ / *n*_Ca2+_** |
| --- | --- | --- | --- | --- | --- | --- |
| 1 | 0 | 180 | 0 | 1 | 0 | 0 |
| 2 | 1 | 181 | 37.5 | 0.99 | 0.21 | 0.21 |
| 3 | 1 | 182 | 37.5 | 0.99 | 0.41 | 0.42 |
| 4 | 1 | 183 | 37.5 | 0.98 | 0.61 | 0.63 |
| 5 | 1 | 184 | 37.5 | 0.98 | 0.82 | 0.83 |
| 6 | 1 | 185 | 37.5 | 0.97 | 1.01 | 1.04 |
| 7 | 1 | 186 | 37.5 | 0.97 | 1.21 | 1.25 |
| 8 | 1 | 187 | 37.5 | 0.96 | 1.4 | 1.46 |
| 9 | 1 | 188 | 37.5 | 0.96 | 1.6 | 1.67 |
| 10 | 1 | 189 | 37.5 | 0.95 | 1.79 | 1.88 |
| 11 | 1 | 190 | 37.5 | 0.95 | 1.97 | 2.08 |
| 12 | 1 | 191 | 37.5 | 0.94 | 2.16 | 2.29 |
| 13 | 1 | 192 | 37.5 | 0.94 | 2.34 | 2.5 |
| 14 | 1 | 193 | 37.5 | 0.93 | 2.53 | 2.71 |
| 15 | 1 | 194 | 150 | 0.93 | 3.29 | 3.54 |
| 16 | 1 | 195 | 150 | 0.92 | 4.04 | 4.38 |
| 17 | 1 | 196 | 150 | 0.92 | 4.78 | 5.21 |
| 18 | 1 | 197 | 150 | 0.91 | 5.52 | 6.04 |
| 19 | 1 | 198 | 150 | 0.91 | 6.25 | 6.88 |
| 20 | 1 | 199 | 150 | 0.9 | 6.97 | 7.71 |
| 21 | 2 | 201 | 150 | 0.9 | 8.4 | 9.38 |
| 22 | 2 | 203 | 150 | 0.89 | 9.79 | 11.04 |
| 23 | 4 | 207 | 150 | 0.87 | 12.5 | 14.38 |

**Table D**

| ***n*_PAF_/*n*_Ca2+_** | **T37** | **D55** | **F31** | **K35** | **C43** | **T44** | **T8** | **C36** |
| --- | --- | --- | --- | --- | --- | --- | --- | --- |
| 0 | 122.08 | 126.64 | 117.14 | 116.87 | 114.19 | 113.35 | 112.87 | 117.55 |
| 0.21 | 122 | 126.81 | 117.1 | 116.97 | 114 | 113.28 | 112.93 | 117.67 |
| 0.42 | 121.92 | 126.98 | 117.03 | 117.03 | 113.86 | 113.23 | 112.96 | 117.77 |
| 0.63 | 121.84 | 127.13 | 116.99 | 117.11 | 113.72 | 113.17 | 113.01 | 117.86 |
| 0.83 | 121.78 | 127.24 | 116.95 | 117.16 | 113.6 | 113.12 | 113.03 | 117.93 |
| 1.04 | 121.72 | 127.35 | 116.92 | 117.21 | 113.5 | 113.08 | 113.07 | 117.99 |
| 1.25 | 121.67 | 127.45 | 116.89 | 117.26 | 113.41 | 113.04 | 113.09 | 118.05 |
| 1.46 | 121.62 | 127.55 | 116.86 | 117.3 | 113.32 | 113 | 113.12 | 118.11 |
| 1.67 | 121.58 | 127.63 | 116.83 | 117.34 | 113.23 | 112.97 | 113.15 | 118.17 |
| 1.88 | 121.54 | 127.71 | 116.81 | 117.38 | 113.15 | 112.94 | 113.17 | 118.22 |
| 2.08 | 121.5 | 127.78 | 116.79 | 117.41 | 113.09 | 112.91 | 113.19 | 118.27 |
| 2.29 | 121.47 | 127.84 | 116.78 | 117.44 | 113.04 | 112.89 | 113.2 | 118.3 |
| 2.5 | 121.45 | 127.9 | 116.75 | 117.47 | 112.98 | 112.87 | 113.22 | 118.34 |
| 2.71 | 121.42 | 127.95 | 116.74 | 117.49 | 112.94 | 112.85 | 113.23 | 118.37 |
| 3.54 | 121.35 | 128.11 | 116.69 | 117.57 | 112.78 | 112.79 | 113.29 | 118.47 |
| 4.38 | 121.27 | 128.25 | 116.65 | 117.63 | 112.66 | 112.74 | 113.32 | 118.56 |
| 5.21 | 121.21 | 128.37 | 116.61 | 117.68 | 112.56 | 112.69 | 113.36 | 118.63 |
| 6.04 | 121.17 | 128.45 | 116.58 | 117.72 | 112.47 | 112.66 | 113.39 | 118.68 |
| 6.88 | 121.13 | 128.53 | 116.56 | 117.76 | 112.41 | 112.63 | 113.4 | 118.73 |
| 7.71 | 121.1 | 128.59 | 116.54 | 117.79 | 112.34 | 112.6 | 113.43 | 118.77 |
| 9.38 | 121.1 | 128.59 | 116.55 | 117.79 | 112.34 | 112.6 | 113.42 | 118.77 |
| 11.04 | 121 | 128.76 | 116.49 | 117.88 | 112.19 | 112.53 | 113.47 | 118.88 |
| 14.38 | 120.96 | 128.87 | 116.47 | 117.92 | 112.09 | 112.5 | 113.51 | 118.95 |
| 17.71 | 120.92 | 128.95 | 116.44 | 117.96 | 112.01 | 112.47 | 113.53 | 119 |
| 21.04 | 120.9 | 128.99 | 116.43 | 117.98 | 111.96 | 112.45 | 113.55 | 119.03 |
| 24.38 | 120.87 | 129.04 | 116.41 | 118 | 111.94 | 112.43 | 113.55 | 119.05 |
| 27.71 | 120.86 | 129.06 | 116.41 | 118.01 | 111.91 | 112.42 | 113.57 | 119.07 |

| ***n*_PAF_/*n*_Ca2+_** | **C54** | **D53** | **V52** | **K34** | **C28** | **K42** | **K9** | **D39** |
| --- | --- | --- | --- | --- | --- | --- | --- | --- |
| 0 | 122.48 | 125.85 | 125.53 | 117.45 | 119.3 | 119.84 | 127.13 | 124.77 |
| 0.21 | 122.78 | 125.78 | 125.59 | 117.7 | 119.38 | 119.77 | 127.16 | 124.81 |
| 0.42 | 123.06 | 125.72 | 125.66 | 117.91 | 119.44 | 119.7 | 127.21 | 124.84 |
| 0.63 | 123.32 | 125.67 | 125.72 | 118.12 | 119.52 | 119.63 | 127.25 | 124.88 |
| 0.83 | 123.52 | 125.63 | 125.76 | 118.28 | 119.57 | 119.58 | 127.27 | 124.9 |
| 1.04 | 123.7 | 125.59 | 125.8 | 118.42 | 119.56 | 119.56 | 127.29 | 124.92 |
| 1.25 | 123.88 | 125.56 | 125.84 | 118.56 | 119.5 | 119.5 | 127.32 | 124.95 |
| 1.46 | 124.05 | 125.51 | 125.88 | 118.68 | 119.7 | 119.45 | 127.34 | 124.96 |
| 1.67 | 124.2 | 125.49 | 125.91 | 118.81 | 119.74 | 119.42 | 127.36 | 124.98 |
| 1.88 | 124.34 | 125.46 | 125.94 | 118.92 | 119.77 | 119.38 | 127.38 | 125 |
| 2.08 | 124.46 | 125.44 | 125.97 | 119.02 | 119.8 | 119.35 | 127.4 | 125.02 |
| 2.29 | 124.56 | 125.42 | 126 | 119.1 | 119.83 | 119.33 | 127.41 | 125.03 |

**Table E**

| **Residue No.** | **Amino acid** | **PAF** | | **PAFD53S/D55S** | |
| --- | --- | --- | --- | --- | --- |
|  |  | **C**α | **C**β | **C**α | **C**β |
| 1 | ALA | 49.162 | 17.107 |  |  |
| 2 | LYS | 52.189 | 31.708 |  |  |
| 3 | TYR | 53.741 | 39.69 | 53.764 | 39.643 |
| 4 | THR | 58.808 | 67.837 | 58.821 | 67.74 |
| 5 | GLY | 42.066 |  | 42.102 | 34.991 |
| 6 | LYS | 51.644 | 34.997 | 51.603 | 42.613 |
| 7 | CYS | 50.41 | 42.645 | 50.398 | 68.384 |
| 8 | THR | 57.291 | 68.608 | 57.302 | 31.086 |
| 9 | LYS | 57.196 | 31.079 | 57.189 | 59.099 |
| 10 | SER | 59.045 |  |  |  |
| 11 | LYS | 53.362 | 30.55 | 53.31 | 30.598 |
| 12 | ASN | 50.874 | 34.483 | 50.901 | 34.525 |
| 13 | GLU | 52.029 | 33.773 | 52.174 | 32.517 |
| 14 | CYS | 51.114 | 39.542 | 51.127 | 39.486 |
| 15 | LYS | 51.435 | 31.472 | 51.708 | 31.663 |
| 16 | TYR | 52.093 | 38.479 | 52.146 | 38.551 |
| 17 | LYS | 51.694 | 30.894 | 51.84 | 30.133 |
| 18 | ASN | 48.158 | 35.143 | 48.311 | 35.274 |
| 19 | ASP | 54.265 | 37.359 |  |  |
| 20 | ALA | 48.765 | 16.013 | 48.861 | 16.183 |
| 21 | GLY | 42.827 |  | 42.798 |  |
| 22 | LYS | 51.436 | 30.576 | 51.68 | 30.128 |
| 23 | ASP | 52.973 | 38.123 | 53.037 | 38.074 |
| 24 | THR | 59.223 | 67.985 | 59.189 | 67.965 |
| 25 | PHE | 53.597 | 39.245 | 53.644 | 39.241 |
| 26 | ILE | 56.844 | 39.475 | 57.036 | 39.506 |
| 27 | LYS | 55.166 | 29.58 | 55.201 | 29.593 |
| 28 | CYS | 52.541 | 38.587 | 53.957 | 38.484 |
| 30 | LYS | 53.497 | 30.09 | 53.539 | 30.131 |
| 31 | PHE | 54.044 | 37.643 | 54.085 | 37.631 |
| 32 | ASP | 54.793 | 37.892 |  | 37.931 |
| 33 | ASN | 51.362 | 34.754 | 51.397 | 34.822 |
| 34 | LYS | 52.148 | 32.813 | 52.559 | 32.796 |
| 35 | LYS | 52.155 | 30.775 |  | 38.731 |
| 36 | CYS | 53.356 | 38.703 |  |  |
| 37 | THR | 60.478 | 68.235 | 60.573 |  |
| 38 | LYS | 53.865 | 32.499 | 53.899 | 32.522 |
| 39 | ASP | 54.033 | 37.454 | 52.668 | 37.403 |
| 40 | ASN | 52.975 | 34.18 | 53.02 | 34.177 |
| 41 | ASN | 49.368 | 36.398 |  | 36.351 |
| 42 | LYS | 54.55 | 30.627 | 54.574 | 30.609 |
| 43 | CYS | 50.891 |  | 51.063 | 47.219 |
| 44 | THR | 57.308 | 69.607 | 57.375 | 69.497 |
| 45 | VAL | 58.069 | 33.384 | 58.057 | 33.248 |
| 46 | ASP | 49.255 | 40.051 | 49.43 | 40.094 |
| 47 | THR | 60.559 | 66.582 | 60.713 | 66.315 |
| 48 | TYR | 57.619 | 35.817 | 57.59 | 35.765 |
| 49 | ASN | 49.231 | 34.484 | 49.245 | 35.79 |
| 50 | ASN | 51.663 | 34.202 | 51.695 | 34.292 |
| 51 | ALA | 49.603 | 17.688 | 49.733 | 17.643 |
| 52 | VAL | 57.894 | 32.369 | 57.939 | 32.397 |
| 53 | ASP | 50.331 | 40.762 |  |  |
| 54 | CYS | 51.047 | 41.549 | 53.504 | 41.804 |
| 55 | ASP | 52.776 | 39.595 |  |  |

**Table F**

| Number of simulation set | % |  |  | % |  |
| --- | --- | --- | --- | --- | --- |
|  |  |  |  |  |  |
| 2. |  |  |  |  |  |
| PAF + 1 Ca^2+^ |  |  | PAF^D53S/D55S^ + 1 Ca^2+^ |  |  |
| Asp53 | 98.64 |  | Asp32 | 6.58 |  |
| Asp55 | 97.47 |  | Ser55-CTER | 0.27 |  |
| Asp55-CTER | 98.61 |  |  |  |  |
|  |  |  |  |  |  |
| 3. |  |  |  |  |  |
| PAF + 10 Ca^2+^ |  |  | PAF^D53S/D55S^ + 10 Ca^2+^ |  |  |
| Asp19 | 19.50 |  | Asp19 | 20.05 |  |
| Asp23 | 10.80 |  | Asp23 | 4.91 |  |
| Asp32 | 14.54 |  | Asp32 | 29.03 |  |
| Asp39 | 37.02 |  | Asp39 | 2.25 |  |
| Asp53 | 61.13 |  | Ser53 | 0.01 |  |
| Asp55 | 40.03 |  | Ser55 | 0.04 |  |
| Asp55-CTER | 99.40 |  | Ser55-CTER | 14.04 |  |
|  |  |  |  |  |  |
| 4. |  |  |  |  |  |
| PAF + 1 Mg^2+^ |  |  |  |  |  |
| Asp53 | 0.03 |  |  |  |  |
| Asp55 | 0.02 |  |  |  |  |
|  |  |  |  |  |  |
| PAF + 1 Ca^2+^ + 1 Mg^2+^ |  |  |  |  |  |
| Ca^2+^ |  |  | Mg^2+^ |  |  |
| Asp53 | 58.79 |  |  |  |  |
| Asp55 | 68.09 |  |  |  |  |
| Asp55-CTER | 85.45 |  |  |  |  |
|  |  |  |  |  |  |
| PAF + 2 Ca^2+^ |  |  |  |  |  |
| Ca^2+^ - 1 |  |  | Ca^2+^ - 2 |  |  |
| Asp53 | 89.08 |  | Asp19 | 3.05 |  |
| Asp55 | 79.95 |  |  |  |  |
| Asp55-CTER | 80.02 |  |  |  |  |

**Figures**


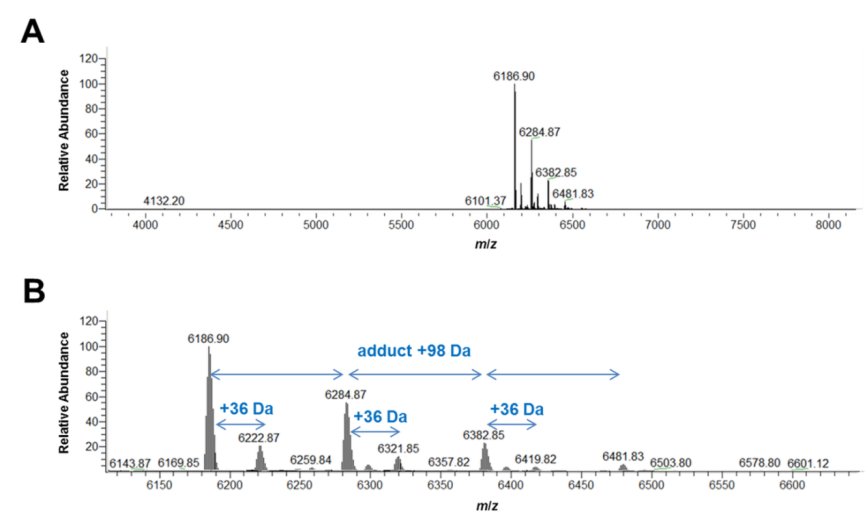


**Figure A**

**
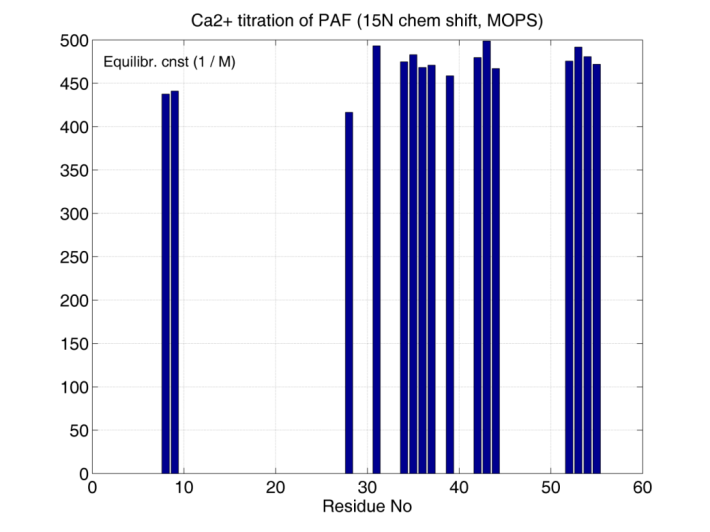
**

**Figure B**


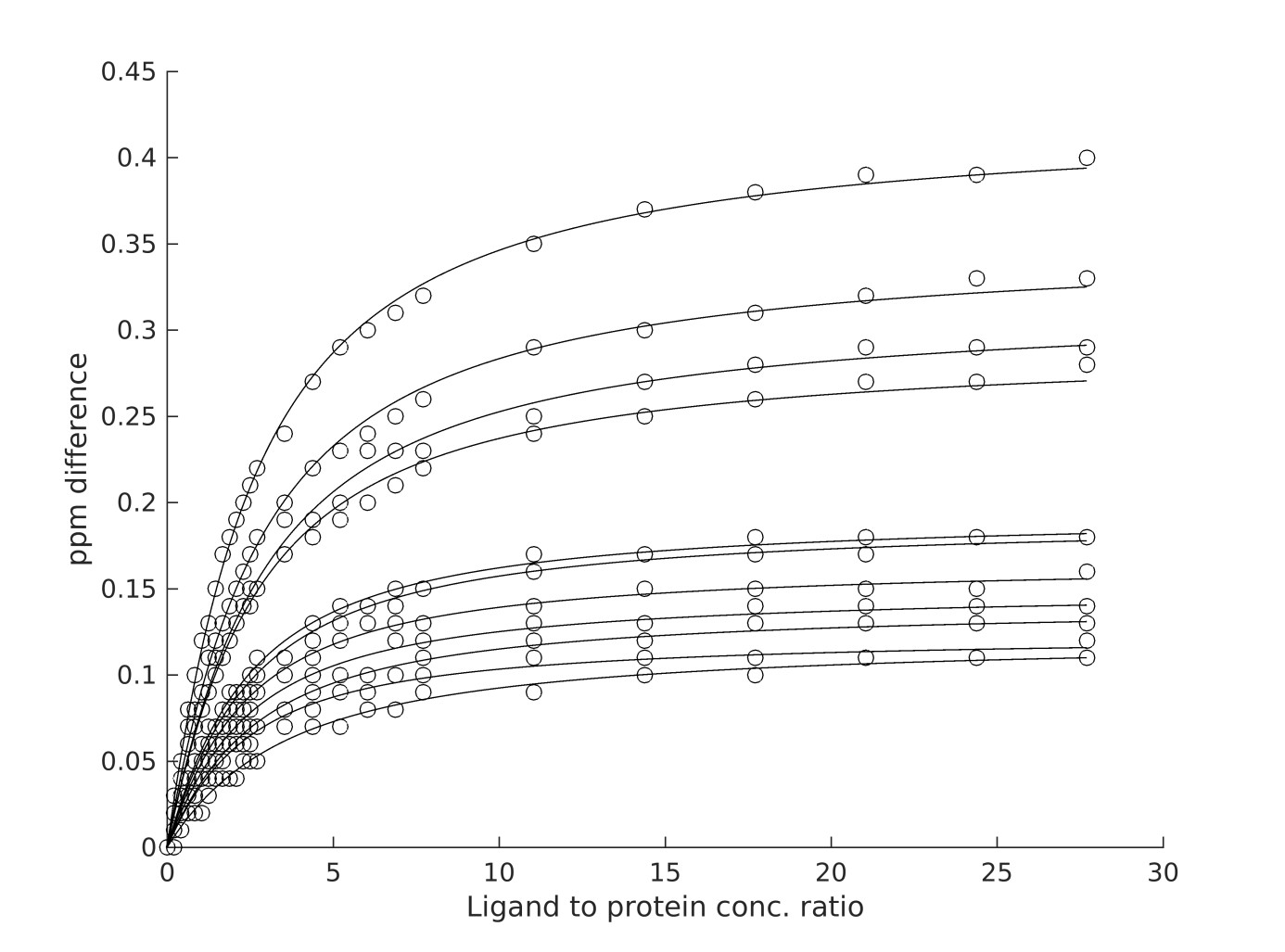


**Figure C**


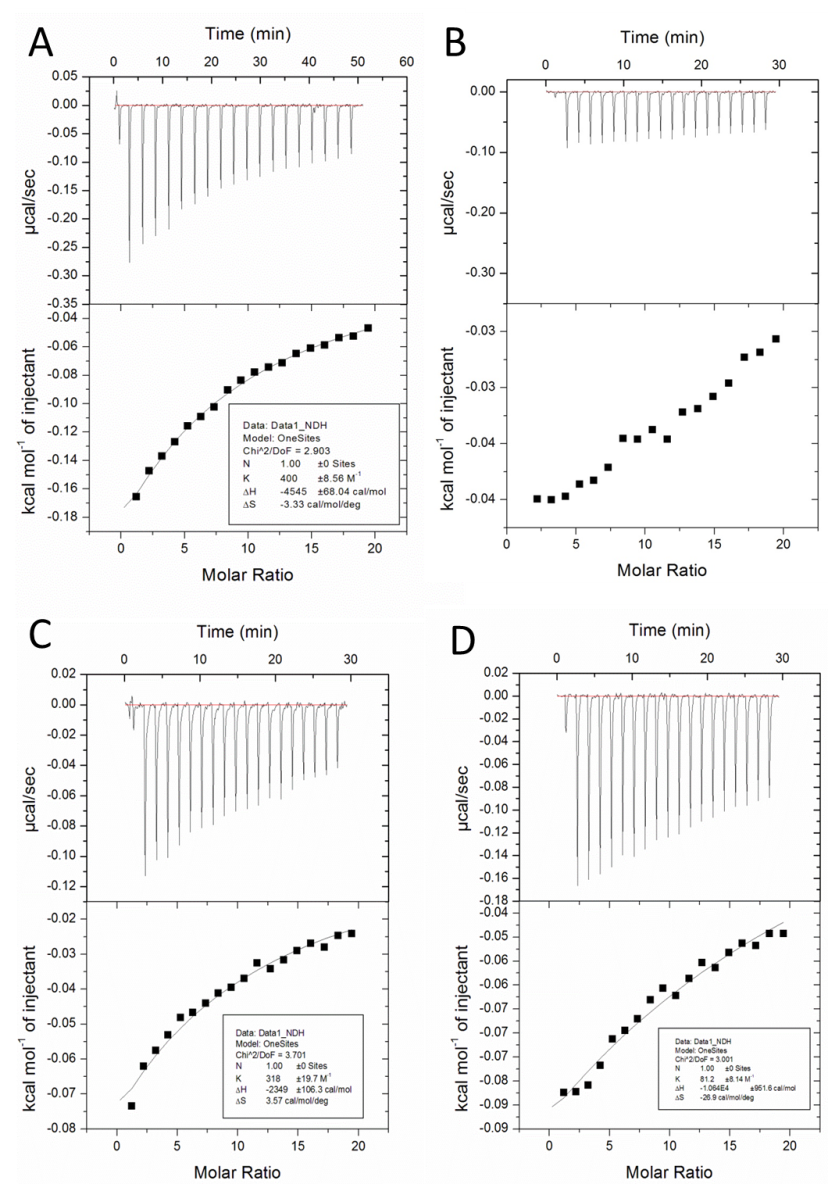


**Figure D**


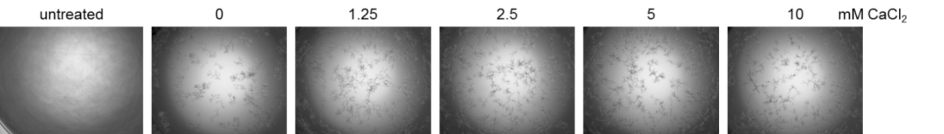


**Figure E**


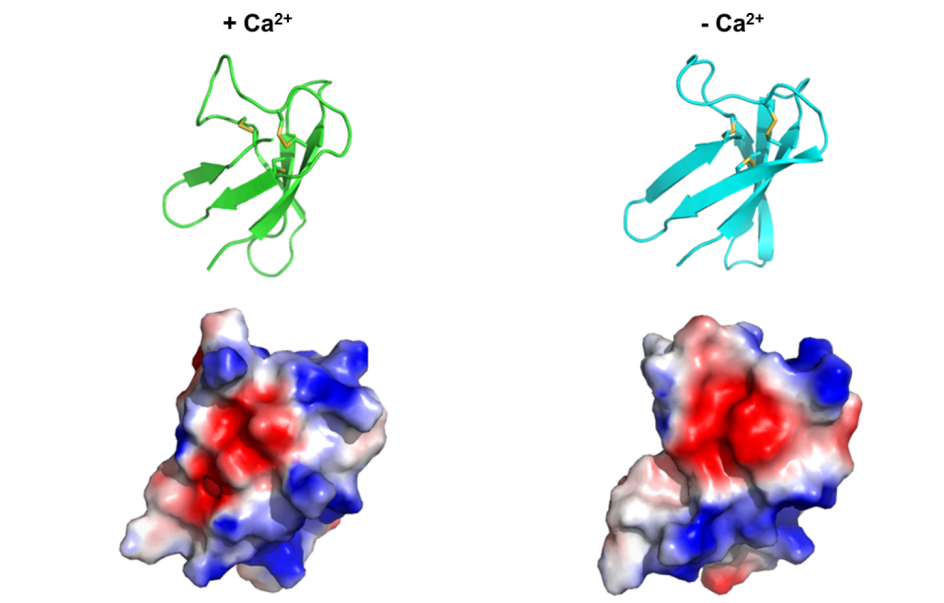


**Figure F**


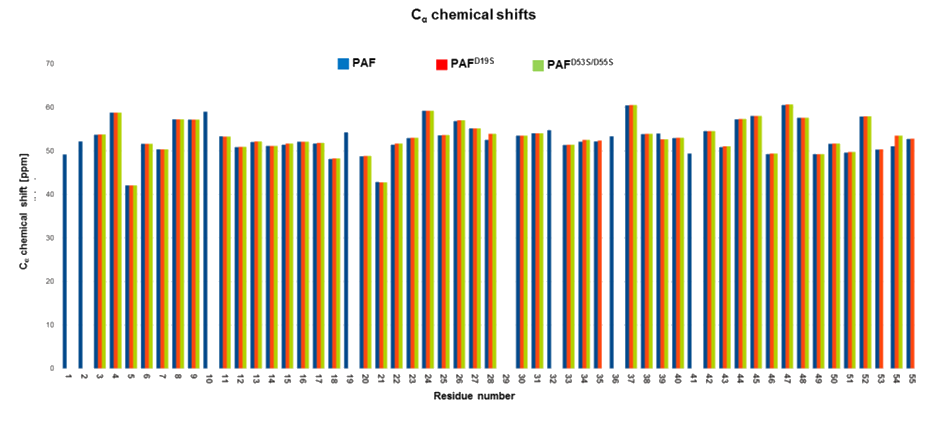


**Figure G**


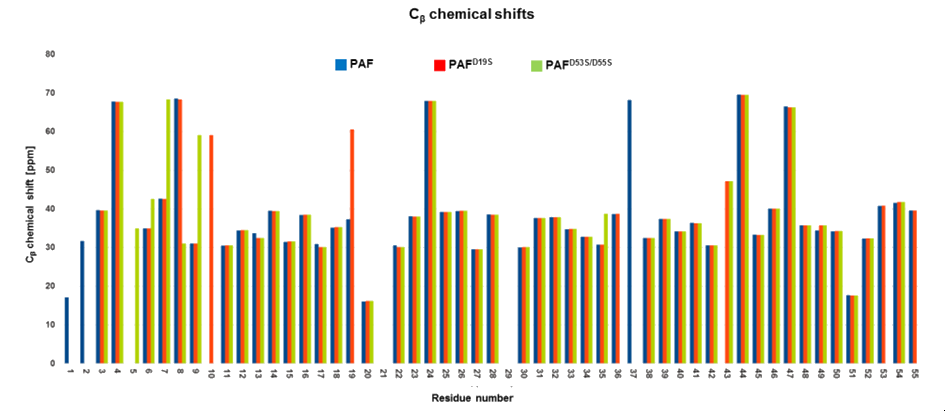


**Figure H**


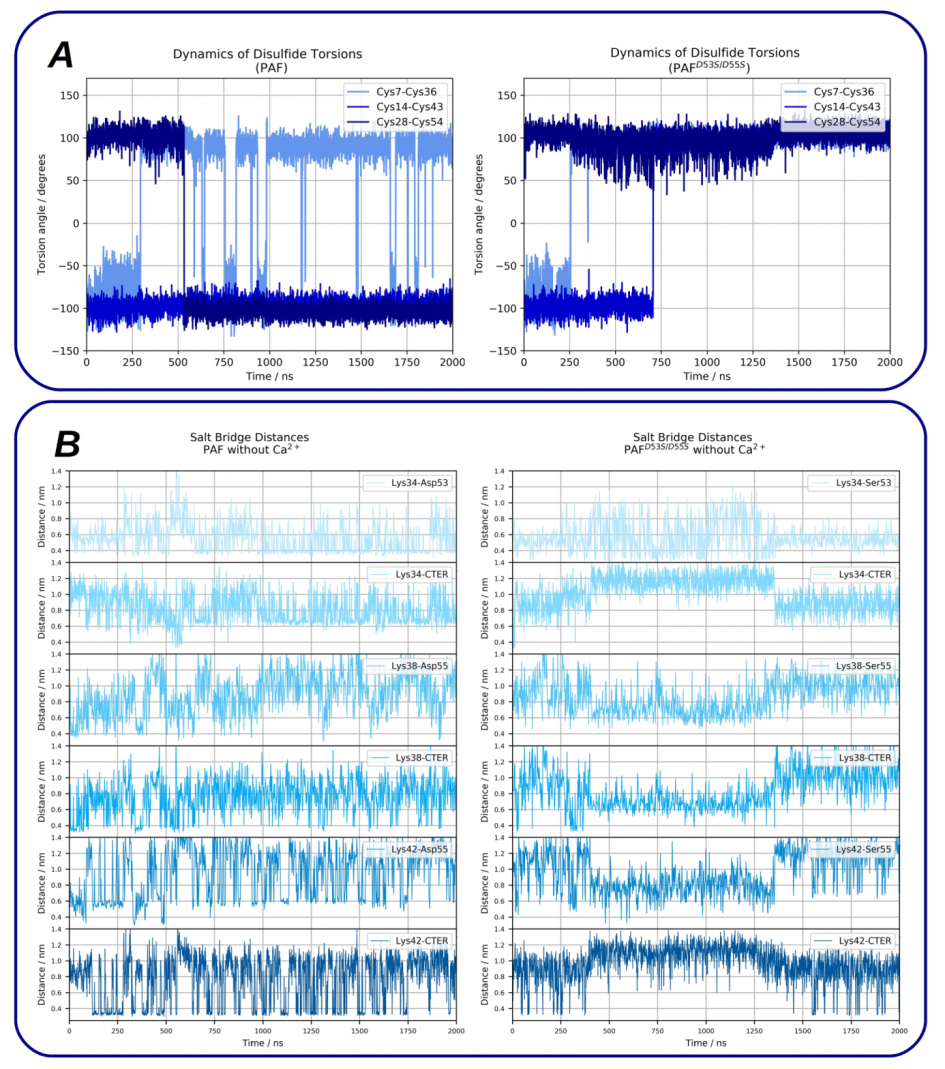


**Figure I**


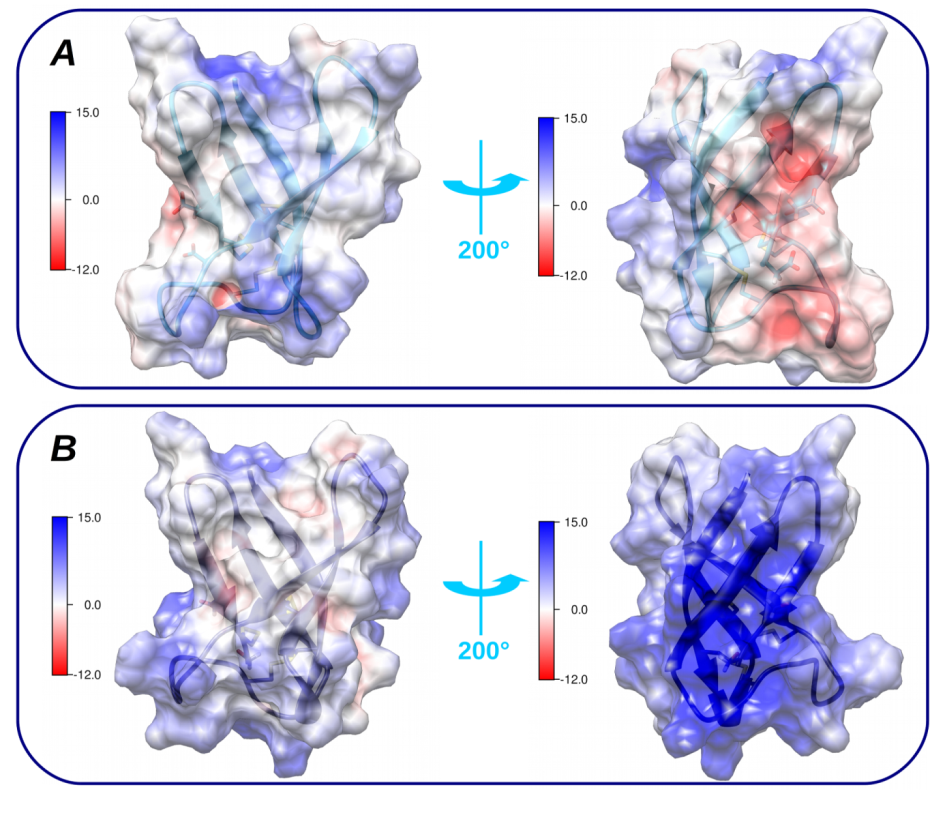


**Figure J**


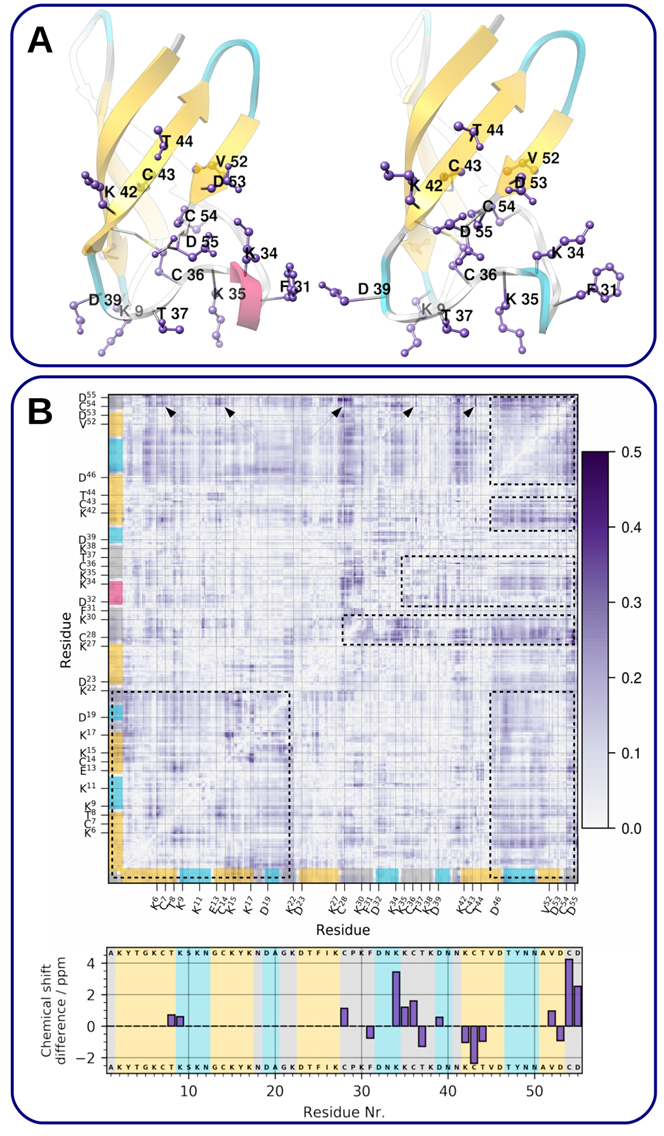


**Figure K**


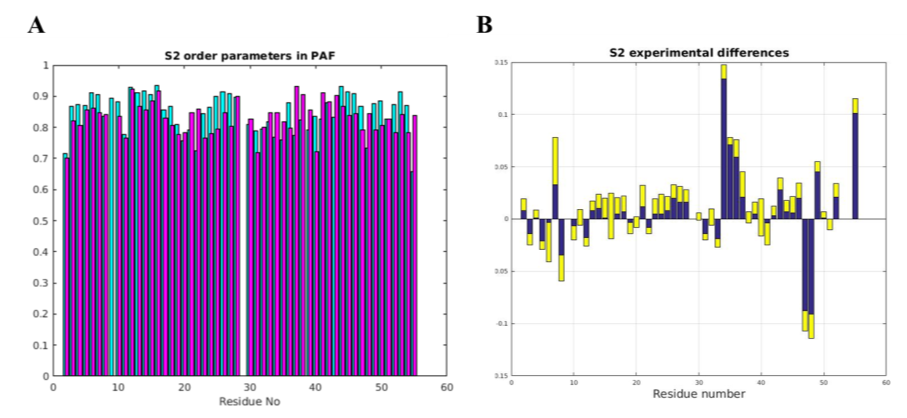


**Figure L**


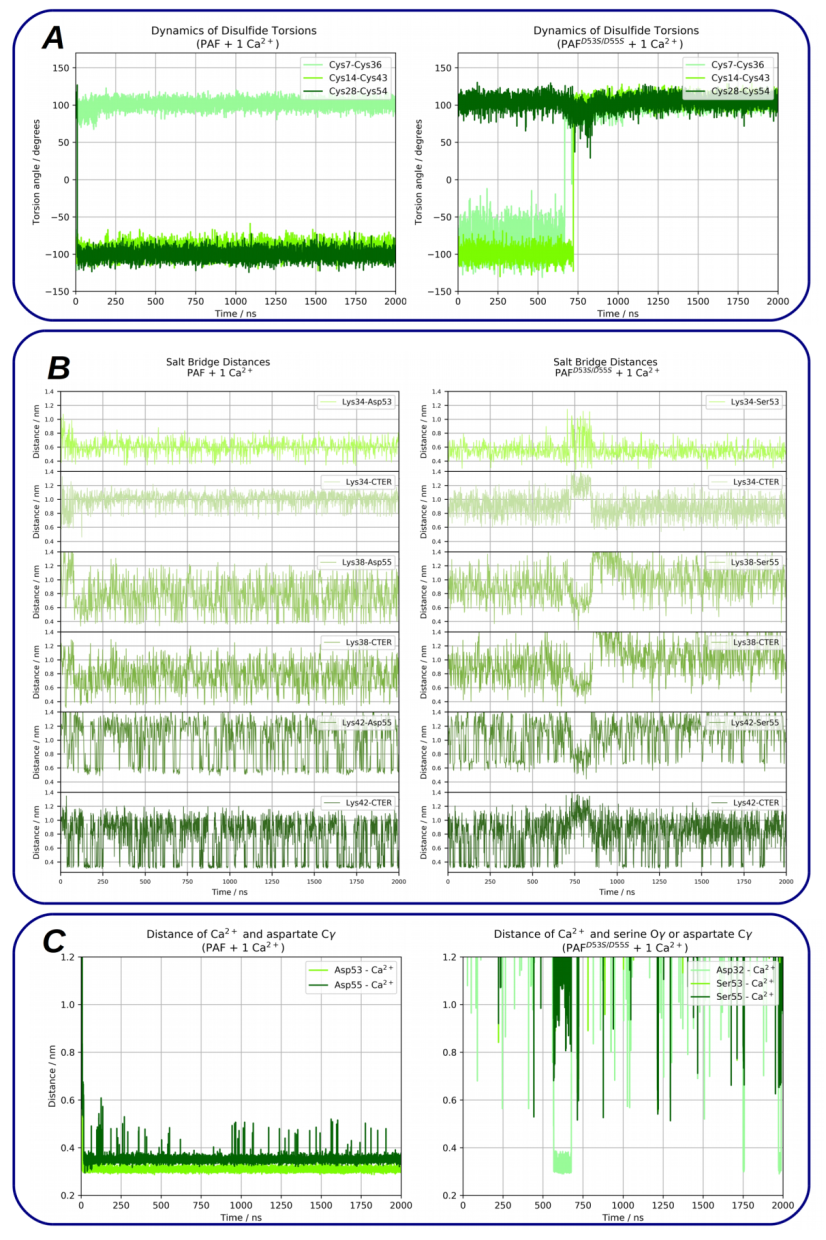


**Figure M**


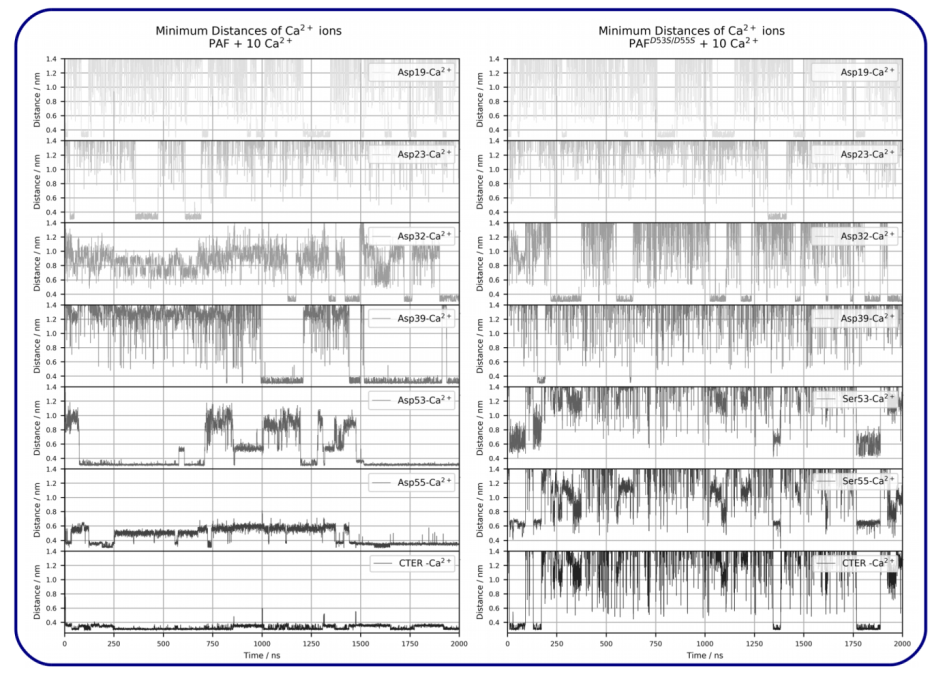


**Figure N**


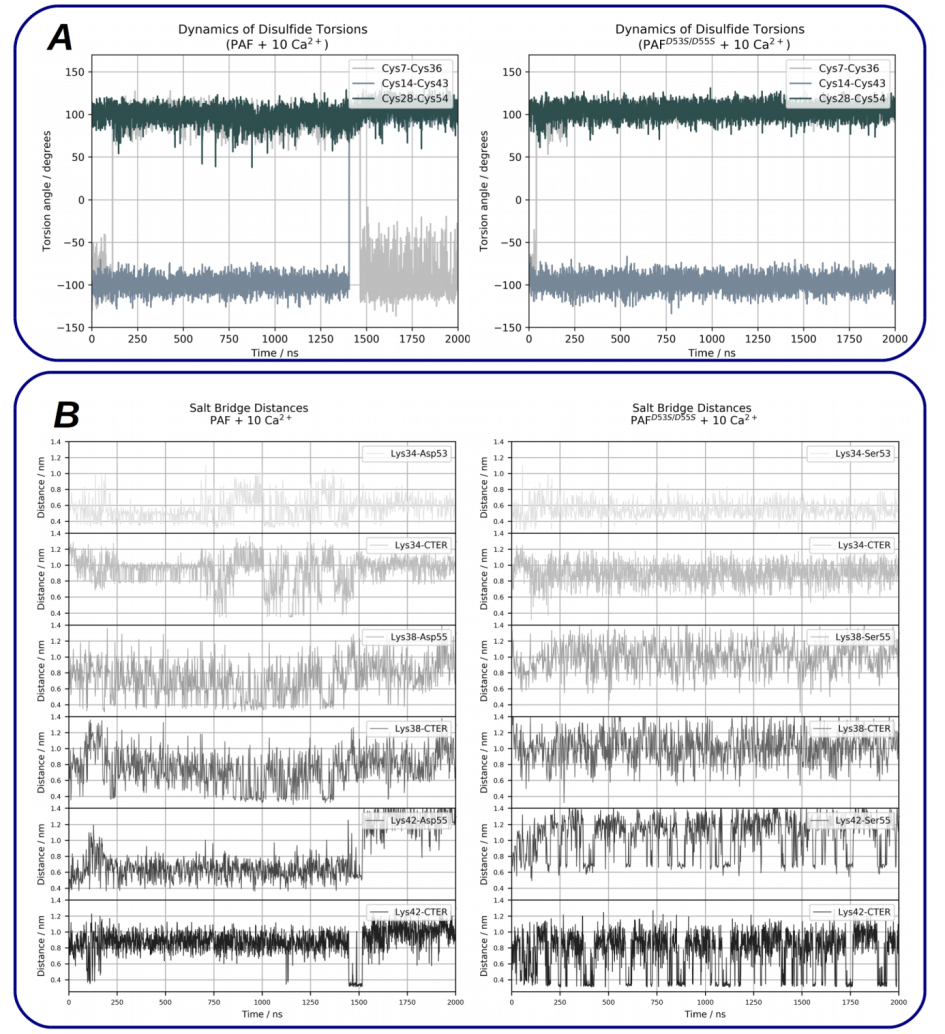


**Figure O**


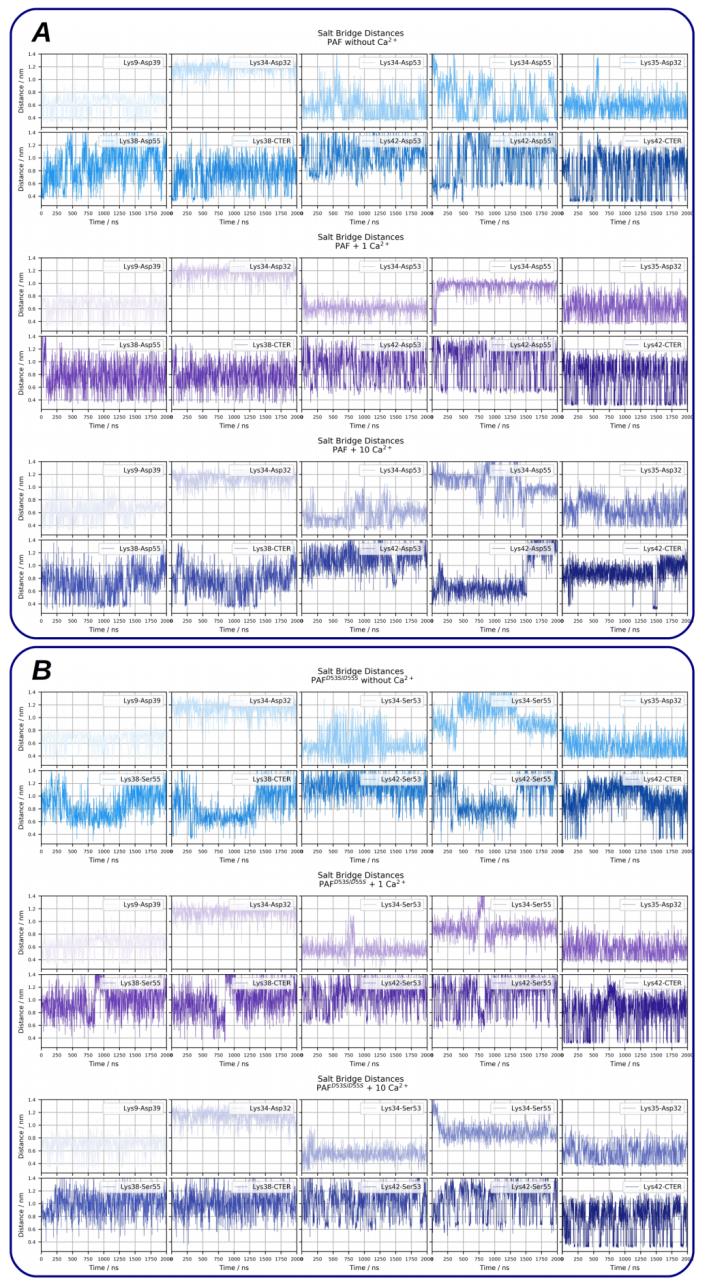


**Figure P**


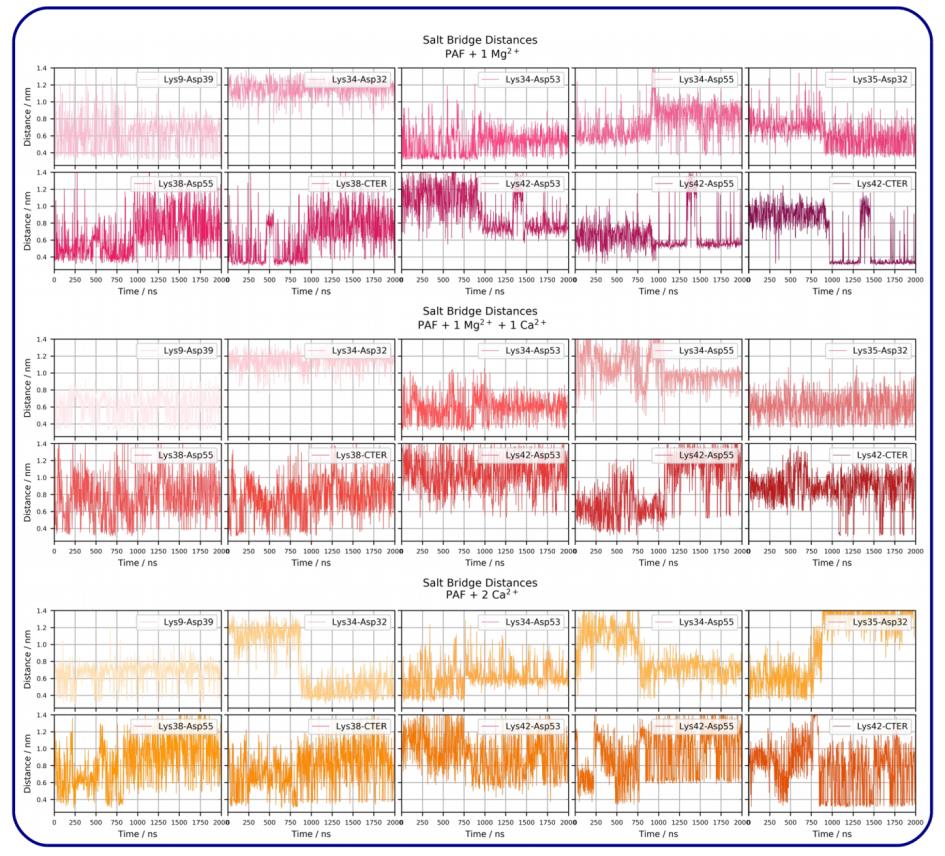


**Figure O**


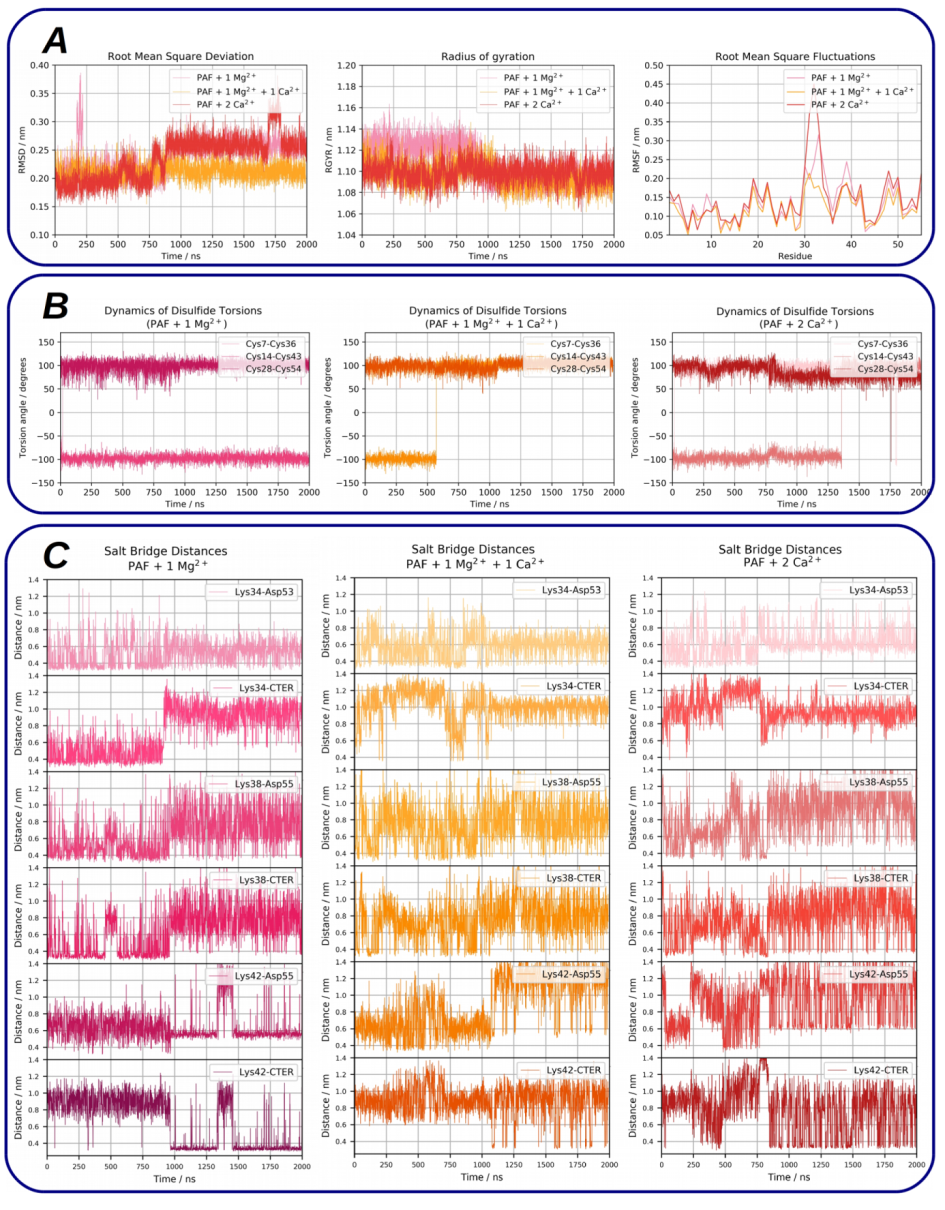


**Figure R**


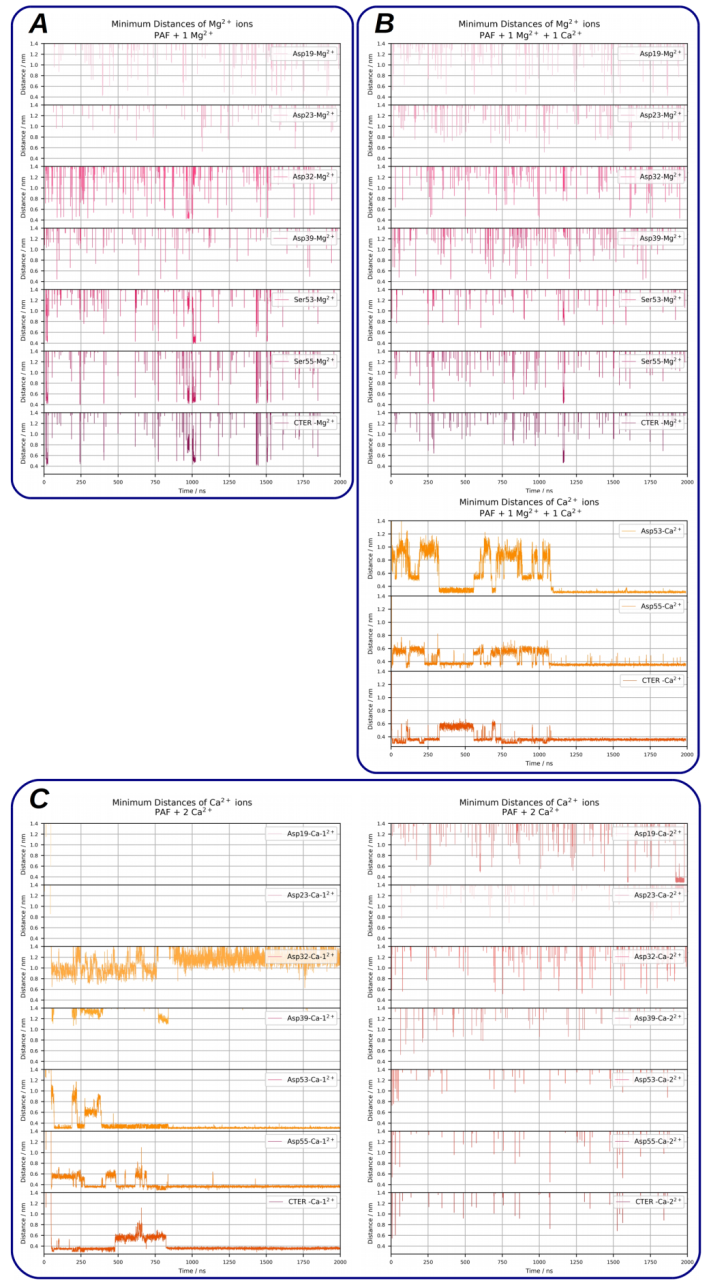


**Figure S**

**
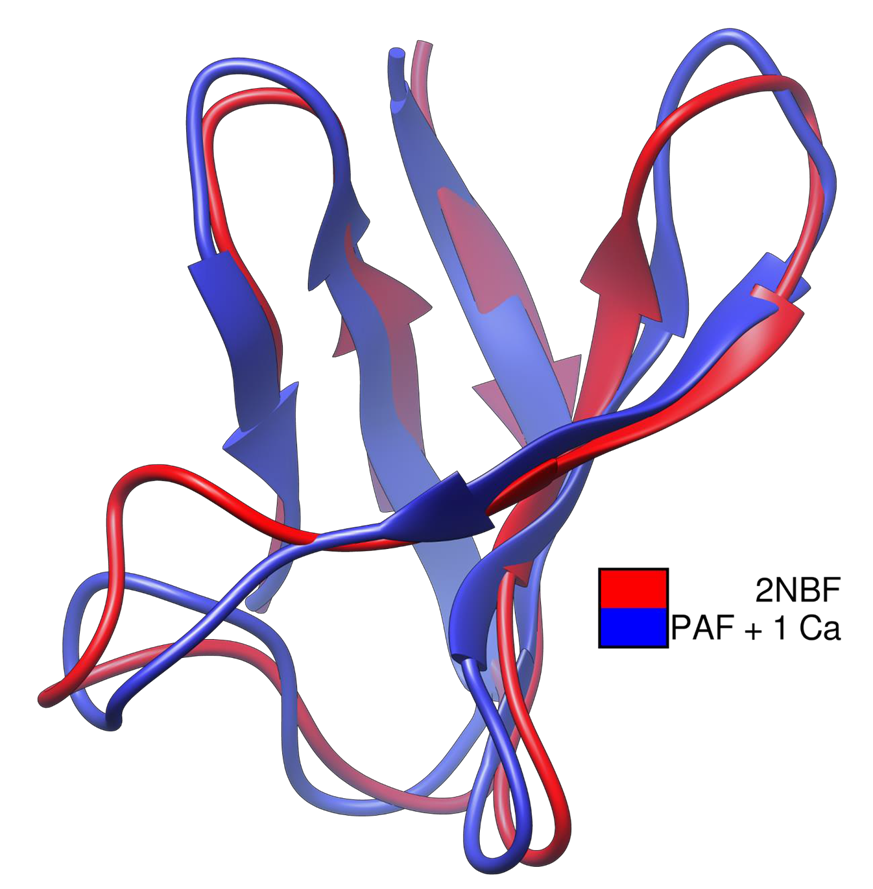
**

**Figure T**
