# Supplementary material for: Calcium binding of the antifungal protein PAF: Structure, dynamics and function aspects by NMR and MD simulations
Source: PLoS One. 2018 Oct 15;13(10):e0204825. doi: 10.1371/journal.pone.0204825 (PMC6188699; doi:10.1371/journal.pone.0204825)
Supplement: S2 File — (PDF) [file pone.0204825.s002.pdf]

PDB ID : 2NBF
RCSB ID : RCSB104657
TITLE   : Structure of calcium-bound form of Penicillium antifungal protein (PAF)
AUTHORS : A.Fizil, G.Batta

The following geometrical and stereochemical features have been calculated for your structure.

CLOSE CONTACTS

==> Distances smaller than 2.2 Angstroms are considered as close contacts for heavy atoms, 1.6 Angstroms for hydrogens.

Chain	Atom	Res	Seq		Chain	Atom	Res	Seq	Mol_ID	Distance
A	SG	CYS	7	-	A	SG	CYS	14	2	Dist = 1.51
A	SG	CYS	7	-	A	SG	CYS	14	3	Dist = 1.88
A	SG	CYS	28	-	A	SG	CYS	36	3	Dist = 2.01
A	SG	CYS	7	-	A	SG	CYS	14	5	Dist = 1.51
A	SG	CYS	7	-	A	SG	CYS	14	8	Dist = 1.53
A	SG	CYS	7	-	A	SG	CYS	14	11	Dist = 1.84
A	SG	CYS	7	-	A	SG	CYS	14	13	Dist = 1.52
A	SG	CYS	7	-	A	SG	CYS	14	18	Dist = 1.81
A	SG	CYS	7	-	A	SG	CYS	14	19	Dist = 1.57

BOND DISTANCES AND ANGLES

Bond and angle checks are performed by first computing the average rms error for all bonds and angles relative to standard values for nucleotide units [L. Clowney et al., Geometric Parameters in Nucleic Acids: Nitrogenous Bases, J.Am.Chem.Soc. 1996, 118, 509-518; A. Gelbin et al., Geometric Parameters in Nucleic Acids: Sugar and Phosphate Constituents, J.Am.Chem.Soc. 1996, 118, 519-529] and amino acid units [R.A. Engh and R. Huber, Structure quality and target parameters, International Tables for Crystallography, Volume F, 2001, 382-392]. Any bond or angle which deviates from the dictionary values by more than six times this computed rms error is identified as an outlier.

==> Covalent Bond Lengths:

The overall RMS deviation for covalent bonds relative to the standard dictionary is   0.005 Angstroms

All covalent bonds lie within a 6.0*RMSD range about the standard dictionary values.

==> Covalent Angle Values:

The overall RMS deviation for covalent angles relative to the standard dictionary is   0.7 degrees.

All covalent bond angles lie within a 6.0*RMSD range about the standard dictionary values.

TORSION ANGLES

The torsion angle distributions have been checked. To view these reports, please refer to the ADIT Validation Server at http://deposit.pdb.org/validate.

==> The following table contains a list of torsion angles outside the expected Ramachandran regions [GJ. Kleywegt and TA. Jones, PHI/PSI-chology: Ramachandran Revisited, Structure 1996, 4, 1395 - 1400].

Residue	Chain	Sequence	Model	PSI	PHI
LYS	A	2	1	105.37	63.37
CYS	A	7	1	−168.91	−110.99
LYS	A	11	1	12.88	−141.07
ASN	A	12	1	114.10	64.11
GLU	A	13	1	96.62	−173.03
TYR	A	16	1	−170.33	−170.58
ALA	A	20	1	−59.14	−178.48
ASP	A	23	1	97.51	−59.20
CYS	A	28	1	79.23	−115.53
ASN	A	49	1	22.72	−149.28
ASN	A	50	1	88.47	51.77
ALA	A	51	1	104.20	−169.61
ASP	A	53	1	53.87	−112.37
CYS	A	7	2	−169.20	−107.91
SER	A	10	2	−74.69	−49.74
ASN	A	12	2	110.05	62.91
GLU	A	13	2	95.69	−169.29
ALA	A	20	2	−42.42	−179.40
ASP	A	23	2	94.47	−61.41
LYS	A	27	2	−165.88	−61.91
CYS	A	28	2	79.89	−110.17
ASN	A	49	2	23.51	−156.37
ASN	A	50	2	94.93	57.35
ALA	A	51	2	116.55	−170.12
ASP	A	53	2	68.52	−100.96
LYS	A	2	3	158.87	63.85
LYS	A	11	3	21.54	−154.22
ASN	A	12	3	115.28	64.82
GLU	A	13	3	107.31	−179.51
TYR	A	16	3	−169.64	−165.81
ALA	A	20	3	−43.16	−178.41
ASP	A	23	3	93.55	−63.52
LYS	A	27	3	−179.37	−56.04
THR	A	37	3	19.73	−152.12
THR	A	47	3	44.07	−91.54
ASN	A	49	3	29.36	−164.50
ASN	A	50	3	84.54	53.76
ASP	A	53	3	52.52	−97.64
LYS	A	2	4	147.50	−176.67
CYS	A	7	4	−168.56	−106.91
SER	A	10	4	−74.95	−49.72
ASN	A	12	4	107.26	61.90
GLU	A	13	4	96.97	−166.99
ALA	A	20	4	32.79	72.29
ASP	A	23	4	93.19	−63.38
LYS	A	27	4	−173.34	−59.18
ASN	A	41	4	−169.51	−78.43
ASN	A	49	4	19.38	−150.44
ASN	A	50	4	87.09	51.31
ALA	A	51	4	133.56	−171.69
ASP	A	53	4	58.74	−105.24
CYS	A	7	5	−168.43	−107.24
SER	A	10	5	−74.77	−49.03
ASN	A	12	5	118.04	65.24
GLU	A	13	5	96.43	−178.69
ASP	A	19	5	−69.12	71.92
ALA	A	20	5	27.33	−165.03
CYS	A	28	5	107.94	−49.67
LYS	A	35	5	−169.33	−67.31
THR	A	47	5	45.83	−91.00
ASN	A	49	5	26.58	−154.81
ASN	A	50	5	87.24	52.04
ALA	A	51	5	107.82	−161.85
LYS	A	2	6	105.60	63.37
CYS	A	7	6	−168.06	−114.97
LYS	A	11	6	20.73	−151.34
ASN	A	12	6	111.18	63.27
GLU	A	13	6	85.16	−162.41
ASN	A	18	6	−74.72	−120.18
ASP	A	19	6	−74.97	−56.88
ALA	A	20	6	53.92	−161.80
LYS	A	22	6	105.41	−52.14
ASP	A	23	6	99.80	−56.42

LYS	A	27	6	−171.46	−59.56
ASN	A	41	6	−169.82	−75.02
ASN	A	50	6	71.75	51.57
CYS	A	7	7	−169.21	−107.71
SER	A	10	7	−76.17	−52.14
ASN	A	12	7	123.00	66.73
GLU	A	13	7	93.28	179.36
TYR	A	16	7	−169.34	−174.92
ASP	A	19	7	−71.39	70.86
ALA	A	20	7	32.98	−163.84
LYS	A	27	7	−169.01	−60.26
CYS	A	28	7	74.70	−112.30
ASN	A	41	7	−168.87	−78.05
ASN	A	50	7	88.56	51.57
SER	A	10	8	−75.55	−49.78
ASP	A	19	8	−72.21	70.46
ALA	A	20	8	25.17	−161.13
LYS	A	27	8	−169.64	−61.37
CYS	A	28	8	77.15	−112.40
ASN	A	50	8	80.80	63.46
ALA	A	51	8	−173.59	−178.63
ASP	A	53	8	72.37	−111.23
CYS	A	7	9	−168.08	−111.78
SER	A	10	9	−75.06	−47.24
ASN	A	12	9	117.70	65.60
GLU	A	13	9	95.78	−177.15
ASP	A	19	9	−72.71	70.10
ALA	A	20	9	24.52	−159.86
LYS	A	27	9	−168.70	−61.58
CYS	A	28	9	78.38	−111.51
CYS	A	36	9	107.98	−54.97
ASN	A	41	9	−169.17	−79.06
ASN	A	50	9	81.53	64.24
CYS	A	7	10	−168.75	−108.72
SER	A	10	10	−76.82	−50.38
ASN	A	12	10	125.82	66.73
GLU	A	13	10	85.92	−177.69
TYR	A	16	10	−169.98	−170.40
ASN	A	18	10	−75.81	−131.26
ASP	A	19	10	−75.67	−55.06
ALA	A	20	10	−39.94	−149.96
ASP	A	23	10	95.84	−60.47
LYS	A	27	10	178.54	−55.12
LYS	A	35	10	−169.64	−75.15
ASN	A	50	10	70.62	51.82
ASP	A	53	10	53.78	−111.34
CYS	A	7	11	−168.94	−107.60
SER	A	10	11	−74.63	−49.11
ASN	A	12	11	117.71	65.13
GLU	A	13	11	94.23	−178.14
TYR	A	16	11	−169.54	−163.59
ASP	A	19	11	32.48	−97.99
ALA	A	20	11	47.11	73.31
LYS	A	22	11	103.18	−51.80
CYS	A	28	11	78.40	−111.51
LYS	A	34	11	22.83	−153.45
ASN	A	50	11	83.82	51.39
ASP	A	53	11	55.75	−95.86
CYS	A	7	12	−169.73	−100.49
LYS	A	11	12	23.41	−142.20
ASN	A	12	12	120.44	65.80
GLU	A	13	12	102.96	179.98
ASP	A	19	12	−72.82	−75.42
LYS	A	22	12	100.33	−58.46
ASP	A	23	12	94.39	−61.67
LYS	A	27	12	177.32	−56.05
LYS	A	34	12	26.38	−153.53
LYS	A	35	12	170.27	−51.74
THR	A	37	12	15.03	−140.12
ASN	A	49	12	26.79	−153.35
ASN	A	50	12	81.92	51.77
ALA	A	51	12	93.79	−160.24
ASP	A	53	12	53.87	−108.44
CYS	A	7	13	−168.39	−115.96
SER	A	10	13	−74.95	−49.46

ASN	A	12	13	120.64	66.04
GLU	A	13	13	93.49	−179.94
ASN	A	18	13	−39.71	−130.48
ASP	A	19	13	30.65	−95.67
ALA	A	20	13	48.38	72.66
LYS	A	22	13	103.65	−50.75
ASP	A	23	13	94.23	−64.25
LYS	A	27	13	−168.60	−68.55
CYS	A	28	13	70.29	−114.36
LYS	A	35	13	168.82	−49.44
ASN	A	49	13	22.32	−140.64
ASN	A	50	13	83.78	51.67
ALA	A	51	13	109.74	−165.13
ASP	A	53	13	56.45	−102.08
CYS	A	7	14	−169.17	−109.69
SER	A	10	14	−74.99	−50.39
ASN	A	12	14	111.02	63.24
GLU	A	13	14	101.08	−173.40
ASN	A	18	14	−41.27	−137.19
ASP	A	19	14	30.28	−95.50
ALA	A	20	14	48.48	73.25
LYS	A	22	14	103.66	−50.83
ASP	A	23	14	98.21	−63.75
LYS	A	27	14	−169.75	−59.76
CYS	A	28	14	79.28	−110.96
PHE	A	31	14	−169.94	−105.31
ASP	A	32	14	−39.76	−131.23
ASN	A	41	14	−169.77	−79.08
ASN	A	49	14	21.69	−141.79
ASN	A	50	14	95.49	57.53
ALA	A	51	14	128.17	−172.51
ASP	A	53	14	64.07	−111.73
LYS	A	11	15	14.03	−144.09
ASN	A	12	15	117.82	64.76
GLU	A	13	15	109.72	179.46
TYR	A	16	15	−169.52	−174.54
ASN	A	18	15	−71.87	−116.41
ASP	A	19	15	−74.91	−72.90
LYS	A	22	15	105.05	−56.48
ASP	A	23	15	94.13	−61.77
LYS	A	27	15	175.60	−54.54
CYS	A	28	15	77.38	−111.71
LYS	A	35	15	−174.48	−66.27
THR	A	37	15	14.52	−145.14
ASN	A	41	15	−173.52	−67.57
THR	A	47	15	46.10	−89.98
ASN	A	49	15	24.21	−154.99
ASN	A	50	15	83.52	51.49
ASP	A	53	15	56.00	−115.49
LYS	A	2	16	150.73	63.27
CYS	A	7	16	−168.88	−110.73
ASN	A	12	16	109.48	62.65
GLU	A	13	16	92.60	−169.68
TYR	A	16	16	−171.75	−172.56
ALA	A	20	16	−48.63	−178.53
ASP	A	23	16	94.35	−63.55
LYS	A	27	16	179.05	−55.15
CYS	A	28	16	76.78	−110.94
CYS	A	36	16	107.10	−51.23
ASN	A	41	16	−169.67	−78.07
THR	A	47	16	40.34	−92.04
ASN	A	49	16	19.08	−150.55
ASN	A	50	16	96.83	57.87
ALA	A	51	16	133.95	−175.96
ASP	A	53	16	70.40	−108.29
CYS	A	7	17	−169.86	−121.40
SER	A	10	17	86.67	−65.22
ASN	A	12	17	135.86	65.05
GLU	A	13	17	93.04	−178.33
ASP	A	19	17	−73.74	69.61
ALA	A	20	17	27.38	−165.06
LYS	A	27	17	−168.28	−60.72
LYS	A	34	17	20.01	−141.97
ASN	A	41	17	−168.88	−78.76
ASN	A	50	17	78.70	51.61

CYS	A	7	18	−168.47	−107.90
SER	A	10	18	−74.61	−47.97
ASN	A	12	18	115.42	64.53
GLU	A	13	18	92.16	−170.39
ASN	A	18	18	−71.61	−121.96
ASP	A	19	18	−74.97	−73.56
LYS	A	22	18	100.74	−58.27
ASP	A	23	18	92.29	−63.71
LYS	A	27	18	−166.69	−60.91
LYS	A	35	18	−178.73	−57.31
ASN	A	49	18	32.03	−150.56
ASN	A	50	18	79.04	51.87
ASP	A	53	18	57.18	−105.92
CYS	A	7	19	−169.32	−109.84
SER	A	10	19	−75.77	−46.55
ASN	A	12	19	119.04	65.73
GLU	A	13	19	98.25	−179.84
TYR	A	16	19	−169.92	−168.77
ASP	A	19	19	34.55	−96.37
ILE	A	26	19	122.95	−177.57
CYS	A	28	19	107.56	−48.79
LYS	A	35	19	−172.59	−60.69
ASN	A	41	19	−169.94	−70.79
ASN	A	49	19	24.90	−142.60
ASN	A	50	19	87.60	51.79
ALA	A	51	19	105.63	−168.04
CYS	A	7	20	−168.03	−106.42
SER	A	10	20	−75.83	−49.98
ASN	A	12	20	108.58	62.25
GLU	A	13	20	110.73	−175.69
ASN	A	18	20	−74.56	−114.68
ASP	A	19	20	−75.24	−57.15
ALA	A	20	20	24.88	−151.73
LYS	A	22	20	102.74	−52.59
ASP	A	23	20	97.44	−59.20
LYS	A	27	20	178.14	−54.77
CYS	A	28	20	78.11	−110.75
LYS	A	35	20	−178.43	−57.92
ASN	A	41	20	−167.56	−75.16
THR	A	47	20	39.59	−93.35
ASN	A	49	20	16.25	−144.25
ASN	A	50	20	87.02	51.36
ALA	A	51	20	114.75	−163.98
ASP	A	53	20	55.66	−100.29

CHIRALITY

The chirality has been checked. O1P, O2P, and hydrogen atoms which do not follow the convention defined in the IUBMB (Liebecq, C. Compendium of Biochemical Nomenclature and Related Documents, 2nd ed.; Portland Press: London and Chapel Hill, 1992) and IUPAC nomenclature (J.L. Markley, A. Bax, Y. Arata, C.W. Hilbers, R. Kaptein, B.D. Sykes, P.E. Wright and K. Wuthrich, Recommendations for the Presentation of NMR Structures of Proteins and Nucleic Acids, Pure & Appl. Chem., Vol. 70, pp. 117−142, 1998) have been standardized. Any other stereochemical violations are listed below.

none

HYDROGEN NOMENCLATURE

The nomenclature of hydrogens has been checked and if necessary has been standardized to comply with PDB nomenclature.

The nomenclature of hydrogens on the ND2 atoms of Asn residues, the NE2 atoms of Gln, and/or on the NH1 and NH2 atoms of Arg residues has been checked for agreement with the standard for E/Z orientation presented in [J.L. Markley, et al., Recommendations for the Presentation of NMR Structures of Proteins and Nucleic Acids, Pure & Appl. Chem., 1998, 70, 117−142]. These hydrogens and/or the NH1 and NH2 atoms of Arg residues may have been switched accordingly.

Full wwPDB NMR Structure Validation Report (i)

Feb 21, 2018 – 12:39 am GMT

PDB ID	:	2NBF
Title	:	Structure of calcium-bound form of Penicillium antifungal protein (PAF)
Authors	:	Fizil, A.; Batta, G.
Deposited on	:	2016-02-04

This is a Full wwPDB NMR Structure Validation Report for a publicly released PDB entry.

We welcome your comments at *validation@mail.wwpdb.org*
A user guide is available at
https://www.wwpdb.org/validation/2017/NMRValidationReportHelp
with specific help available everywhere you see the (i) symbol.

The following versions of software and data (see references (i)) were used in the production of this report:

Cyrange	:	Kirchner and Güntert (2011)
NmrClust	:	Kelley et al. (1996)
MolProbity	:	4.02b-467
Percentile statistics	:	20171227.v01 (using entries in the PDB archive December 27th 2017)
RCI	:	v_1n_11_5_13_A (Berjanski et al., 2005)
PANAV	:	Wang et al. (2010)
ShiftChecker	:	trunk30686
Ideal geometry (proteins)	:	Engh & Huber (2001)
Ideal geometry (DNA, RNA)	:	Parkinson et al. (1996)
Validation Pipeline (wwPDB-VP)	:	trunk30686

1 Overall quality at a glance (i)

The following experimental techniques were used to determine the structure:
SOLUTION NMR

The overall completeness of chemical shifts assignment is 48%.

Percentile scores (ranging between 0-100) for global validation metrics of the entry are shown in the following graphic. The table shows the number of entries on which the scores are based.

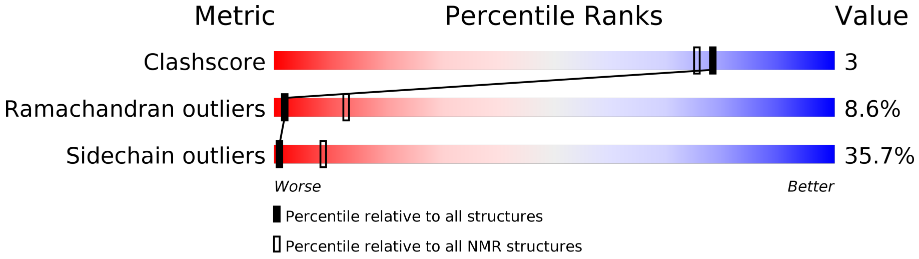

Metric	Whole archive (#Entries)	NMR archive (#Entries)
Clashscore	136279	12091
Ramachandran outliers	132675	10835
Sidechain outliers	132484	10811

The table below summarises the geometric issues observed across the polymeric chains and their fit to the experimental data. The red, orange, yellow and green segments indicate the fraction of residues that contain outliers for >=3, 2, 1 and 0 types of geometric quality criteria. A cyan segment indicates the fraction of residues that are not part of the well-defined cores, and a grey segment represents the fraction of residues that are not modelled. The numeric value for each fraction is indicated below the corresponding segment, with a dot representing fractions <=5%

Mol	Chain	Length	Quality of chain
1	A	55	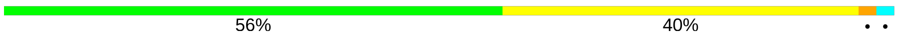

2 Ensemble composition and analysis (i)

This entry contains 20 models. Model 16 is the overall representative, medoid model (most similar to other models). The authors have identified model 1 as representative, based on the following criterion: *closest to the average*.

The following residues are included in the computation of the global validation metrics.

Well-defined (core) protein residues			
**Well-defined core**	**Residue range (total)**	**Backbone RMSD (Å)**	**Medoid model**
1	A:2-A:55 (54)	0.36	16

Ill-defined regions of proteins are excluded from the global statistics.

Ligands and non-protein polymers are included in the analysis.

The models can be grouped into 2 clusters. No single-model clusters were found.

Cluster number	Models
1	1, 2, 3, 4, 6, 7, 8, 9, 10, 11, 12, 13, 14, 15, 16, 17, 18, 20
2	5, 19

3 Entry composition (i)

There is only 1 type of molecule in this entry. The entry contains 850 atoms, of which 418 are hydrogens and 0 are deuteriums.

- Molecule 1 is a protein called Antifungal protein.

Mol	Chain	Residues	Atoms						Trace
			Total	C	H	N	O	S	
1	A	55	850	263	418	75	88	6	0

4 Residue-property plots (i)

4.1 Average score per residue in the NMR ensemble

These plots are provided for all protein, RNA and DNA chains in the entry. The first graphic is the same as shown in the summary in section 1 of this report. The second graphic shows the sequence where residues are colour-coded according to the number of geometric quality criteria for which they contain at least one outlier: green = 0, yellow = 1, orange = 2 and red = 3 or more. Stretches of 2 or more consecutive residues without any outliers are shown as green connectors. Residues which are classified as ill-defined in the NMR ensemble, are shown in cyan with an underline colour-coded according to the previous scheme. Residues which were present in the experimental sample, but not modelled in the final structure are shown in grey.

- Molecule 1: Antifungal protein

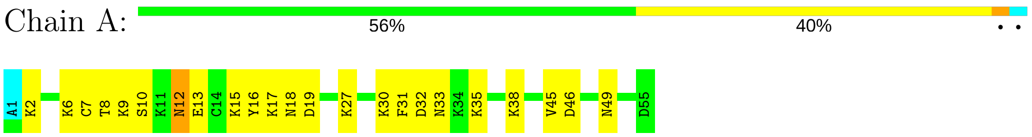

4.2 Scores per residue for each member of the ensemble

Colouring as in section 4.1 above.

4.2.1 Score per residue for model 1

- Molecule 1: Antifungal protein

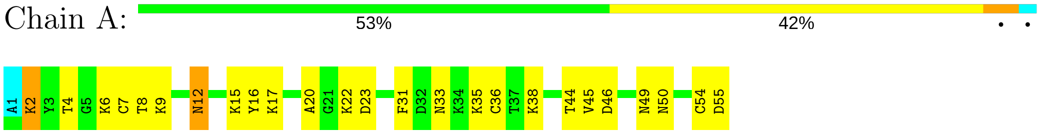

4.2.2 Score per residue for model 2

- Molecule 1: Antifungal protein

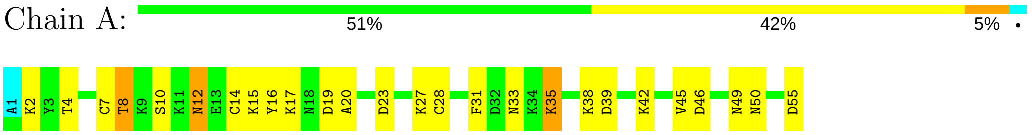

4.2.3 Score per residue for model 3

• Molecule 1: Antifungal protein

Chain A:

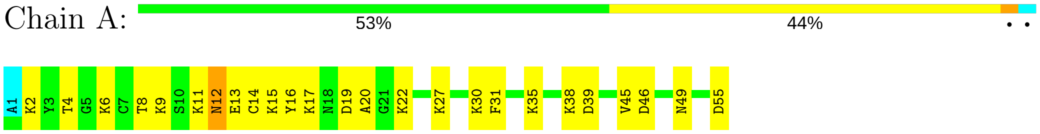

4.2.4 Score per residue for model 4

• Molecule 1: Antifungal protein

Chain A:

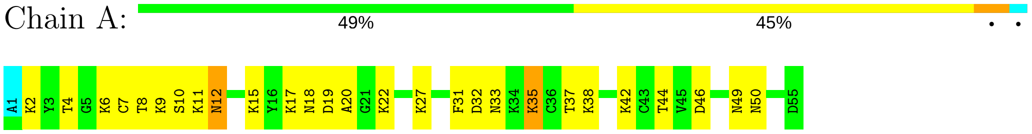

4.2.5 Score per residue for model 5

• Molecule 1: Antifungal protein

Chain A:

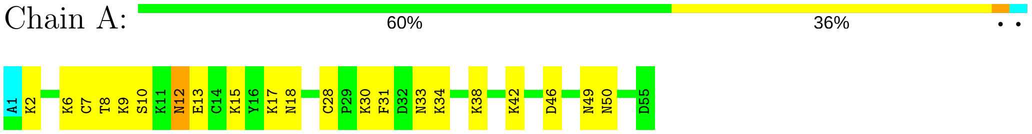

4.2.6 Score per residue for model 6

• Molecule 1: Antifungal protein

Chain A:

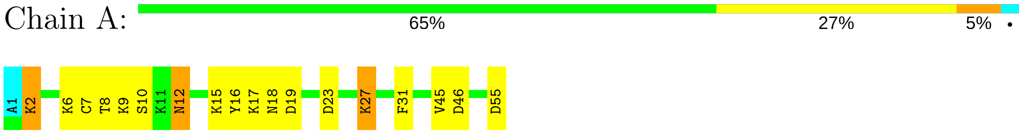

4.2.7 Score per residue for model 7

• Molecule 1: Antifungal protein

Chain A:

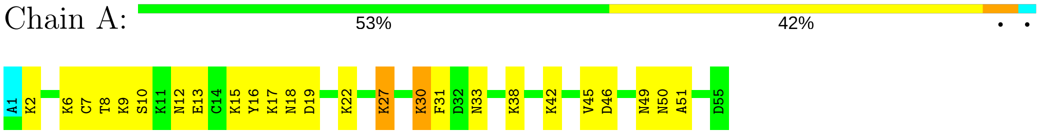

4.2.8 Score per residue for model 8

• Molecule 1: Antifungal protein

Chain A:

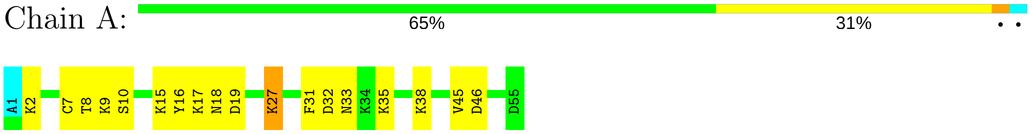

4.2.9 Score per residue for model 9

• Molecule 1: Antifungal protein

Chain A:

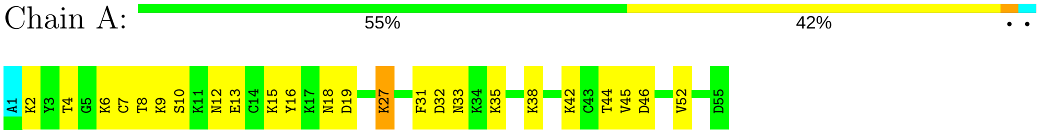

4.2.10 Score per residue for model 10

• Molecule 1: Antifungal protein

Chain A:

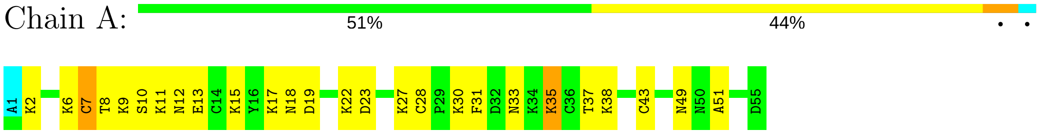

4.2.11 Score per residue for model 11

• Molecule 1: Antifungal protein

Chain A:

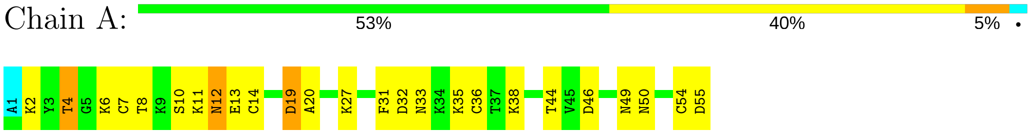

4.2.12 Score per residue for model 12

• Molecule 1: Antifungal protein

Chain A:

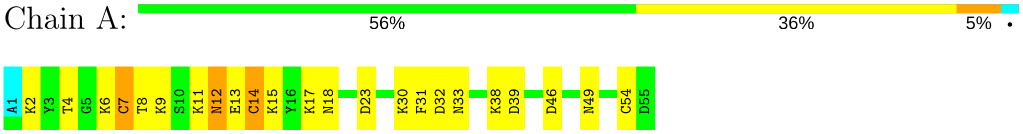

4.2.13 Score per residue for model 13

- Molecule 1: Antifungal protein

Chain A:

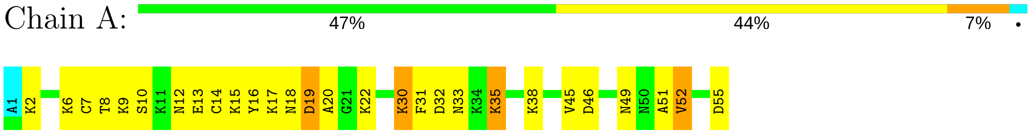

4.2.14 Score per residue for model 14

- Molecule 1: Antifungal protein

Chain A:

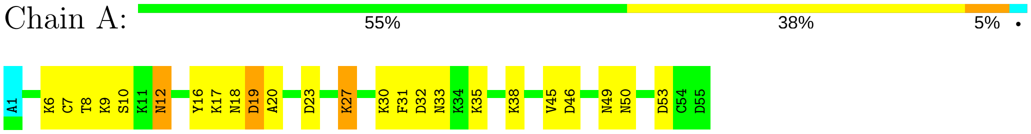

4.2.15 Score per residue for model 15

- Molecule 1: Antifungal protein

Chain A:

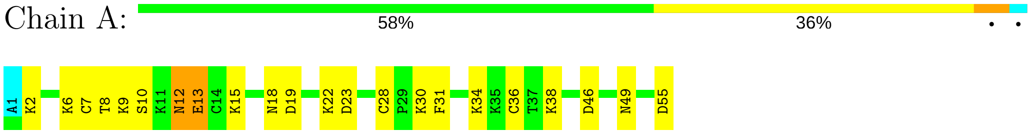

4.2.16 Score per residue for model 16 (medoid)

- Molecule 1: Antifungal protein

Chain A:

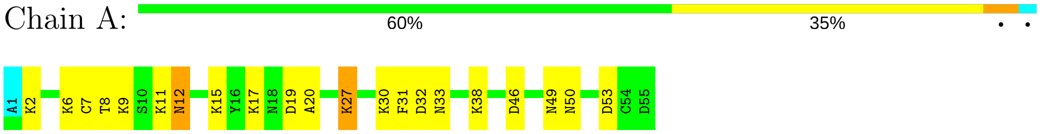

4.2.17 Score per residue for model 17

- Molecule 1: Antifungal protein

Chain A:

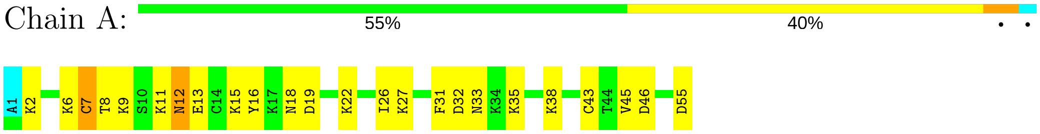

4.2.18 Score per residue for model 18

- Molecule 1: Antifungal protein

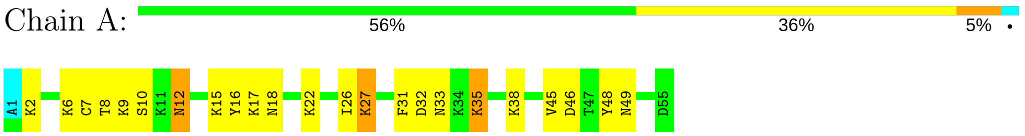

4.2.19 Score per residue for model 19

- Molecule 1: Antifungal protein

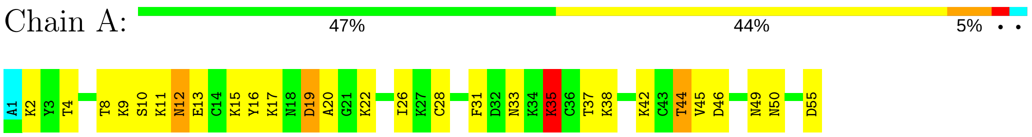

4.2.20 Score per residue for model 20

- Molecule 1: Antifungal protein

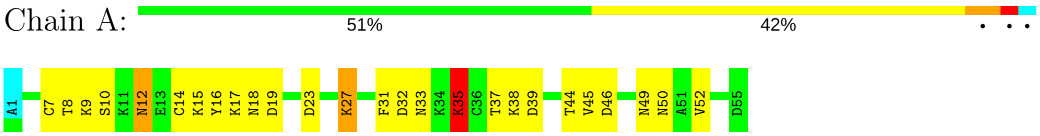

5 Refinement protocol and experimental data overview (i)

The models were refined using the following method: *simulated annealing, torsion angle dynamics*.

Of the 20 calculated structures, 20 were deposited, based on the following criterion: *structures with the lowest energy*.

The following table shows the software used for structure solution, optimisation and refinement.

Software name	Classification	Version
CYANA	structure solution	2.1
ATNOS-CANDID	structure solution	
CYANA	refinement	

The following table shows chemical shift validation statistics as aggregates over all chemical shift files. Detailed validation can be found in section 7 of this report.

Chemical shift file(s)	2nbf_cs.str
Number of chemical shift lists	1
Total number of shifts	350
Number of shifts mapped to atoms	350
Number of unparsed shifts	0
Number of shifts with mapping errors	0
Number of shifts with mapping warnings	0
Assignment completeness (well-defined parts)	48%

No validations of the models with respect to experimental NMR restraints is performed at this time.

6 Model quality (i)

6.1 Standard geometry (i)

There are no covalent bond-length or bond-angle outliers.

There are no bond-length outliers.

There are no bond-angle outliers.

There are no chirality outliers.

There are no planarity outliers.

6.2 Too-close contacts (i)

In the following table, the Non-H and H(model) columns list the number of non-hydrogen atoms and hydrogen atoms in each chain respectively. The H(added) column lists the number of hydrogen atoms added and optimized by MolProbity. The Clashes column lists the number of clashes averaged over the ensemble.

Mol	Chain	Non-H	H(model)	H(added)	Clashes
1	A	427	413	413	3±2
All	All	8540	8260	8260	52

The all-atom clashscore is defined as the number of clashes found per 1000 atoms (including hydrogen atoms). The all-atom clashscore for this structure is 3.

All unique clashes are listed below, sorted by their clash magnitude.

Atom-1	Atom-2	Clash(Å)	Distance(Å)	Models	
				Worst	Total
1:A:28:CYS:SG	1:A:36:CYS:SG	0.95	2.63	15	1
1:A:14:CYS:SG	1:A:54:CYS:SG	0.86	2.73	11	2
1:A:7:CYS:SG	1:A:14:CYS:SG	0.77	2.83	12	1
1:A:4:THR:HG23	1:A:44:THR:HG22	0.60	1.73	11	1
1:A:16:TYR:CZ	1:A:45:VAL:HG11	0.59	2.33	3	4
1:A:35:LYS:HD2	1:A:37:THR:HG23	0.55	1.78	4	1
1:A:36:CYS:SG	1:A:54:CYS:CB	0.52	2.97	11	2
1:A:45:VAL:HG22	1:A:52:VAL:HG23	0.51	1.81	9	2
1:A:8:THR:HG22	1:A:39:ASP:OD1	0.51	2.05	2	1
1:A:16:TYR:CD1	1:A:45:VAL:HG21	0.50	2.41	20	2
1:A:30:LYS:HG3	1:A:51:ALA:HB1	0.48	1.85	13	2
1:A:19:ASP:O	1:A:20:ALA:HB3	0.48	2.09	19	5
1:A:4:THR:HA	1:A:44:THR:HG22	0.48	1.84	9	4
1:A:16:TYR:CE1	1:A:45:VAL:HG11	0.48	2.43	20	4

Continued on next page...

Continued from previous page...

Atom-1	Atom-2	Clash(Å)	Distance(Å)	Models	
				Worst	Total
1:A:14:CYS:HB3	1:A:52:VAL:HG21	0.48	1.84	20	1
1:A:16:TYR:CG	1:A:45:VAL:HG21	0.47	2.44	13	2
1:A:7:CYS:SG	1:A:54:CYS:SG	0.47	3.12	12	1
1:A:4:THR:HG23	1:A:44:THR:CG2	0.47	2.38	11	2
1:A:16:TYR:CE2	1:A:45:VAL:HG11	0.46	2.46	18	1
1:A:16:TYR:CD2	1:A:45:VAL:HG21	0.46	2.45	7	6
1:A:49:ASN:ND2	1:A:51:ALA:HB2	0.45	2.27	10	1
1:A:35:LYS:O	1:A:37:THR:HG23	0.44	2.11	10	3
1:A:7:CYS:SG	1:A:43:CYS:CB	0.43	3.05	17	2
1:A:14:CYS:CB	1:A:52:VAL:HG21	0.42	2.43	20	1

6.3 Torsion angles (i)

6.3.1 Protein backbone (i)

In the following table, the Percentiles column shows the percent Ramachandran outliers of the chain as a percentile score with respect to all PDB entries followed by that with respect to all NMR entries. The Analysed column shows the number of residues for which the backbone conformation was analysed and the total number of residues.

Mol	Chain	Analysed	Favoured	Allowed	Outliers	Percentiles	
1	A	53/55 (96%)	37±2 (69±4%)	12±2 (22±5%)	5±1 (9±2%)	2	13
All	All	1060/1100 (96%)	731 (69%)	238 (22%)	91 (9%)	2	13

All 11 unique Ramachandran outliers are listed below. They are sorted by the frequency of occurrence in the ensemble.

Mol	Chain	Res	Type	Models (Total)
1	A	12	ASN	19
1	A	27	LYS	14
1	A	10	SER	13
1	A	13	GLU	11
1	A	50	ASN	10
1	A	23	ASP	7
1	A	35	LYS	5
1	A	20	ALA	4
1	A	2	LYS	3
1	A	19	ASP	3
1	A	26	ILE	2

6.3.2 Protein sidechains (i)

In the following table, the Percentiles column shows the percent sidechain outliers of the chain as a percentile score with respect to all PDB entries followed by that with respect to all NMR entries. The Analysed column shows the number of residues for which the sidechain conformation was analysed and the total number of residues.

Mol	Chain	Analysed	Rotameric	Outliers	Percentiles
1	A	50/50 (100%)	32±2 (64±4%)	18±2 (36±4%)	1 9
All	All	1000/1000 (100%)	643 (64%)	357 (36%)	1 9

All 36 unique residues with a non-rotameric sidechain are listed below. They are sorted by the frequency of occurrence in the ensemble.

Mol	Chain	Res	Type	Models (Total)
1	A	31	PHE	20
1	A	8	THR	20
1	A	46	ASP	19
1	A	38	LYS	19
1	A	15	LYS	18
1	A	9	LYS	18
1	A	7	CYS	18
1	A	33	ASN	17
1	A	2	LYS	17
1	A	17	LYS	16
1	A	6	LYS	16
1	A	49	ASN	15
1	A	12	ASN	15
1	A	18	ASN	14
1	A	35	LYS	14
1	A	19	ASP	12
1	A	32	ASP	11
1	A	22	LYS	10
1	A	30	LYS	9
1	A	55	ASP	9
1	A	27	LYS	8
1	A	11	LYS	8
1	A	42	LYS	6
1	A	4	THR	4
1	A	28	CYS	4
1	A	14	CYS	4
1	A	39	ASP	3
1	A	53	ASP	2
1	A	10	SER	2

Continued on next page...

Continued from previous page...

Mol	Chain	Res	Type	Models (Total)
1	A	34	LYS	2
1	A	44	THR	2
1	A	52	VAL	1
1	A	13	GLU	1
1	A	48	TYR	1
1	A	23	ASP	1
1	A	26	ILE	1

6.3.3 RNA (i)

There are no RNA molecules in this entry.

6.4 Non-standard residues in protein, DNA, RNA chains (i)

There are no non-standard protein/DNA/RNA residues in this entry.

6.5 Carbohydrates (i)

There are no carbohydrates in this entry.

6.6 Ligand geometry (i)

There are no ligands in this entry.

6.7 Other polymers (i)

There are no such molecules in this entry.

6.8 Polymer linkage issues (i)

There are no chain breaks in this entry.

7 Chemical shift validation (i)

The completeness of assignment taking into account all chemical shift lists is 48% for the well-defined parts and 48% for the entire structure.

7.1 Chemical shift list 1

File name: 2nbf_cs.str

Chemical shift list name: *assigned_chem_shift_list_1*

7.1.1 Bookkeeping (i)

The following table shows the results of parsing the chemical shift list and reports the number of nuclei with statistically unusual chemical shifts.

Total number of shifts	350
Number of shifts mapped to atoms	350
Number of unparsed shifts	0
Number of shifts with mapping errors	0
Number of shifts with mapping warnings	0
Number of shift outliers (ShiftChecker)	2

7.1.2 Chemical shift referencing (i)

The following table shows the suggested chemical shift referencing corrections.

Nucleus	# values	Correction ± precision, *ppm*	Suggested action
$^{13}C_{\alpha}$	0	—	None (insufficient data)
$^{13}C_{\beta}$	0	—	None (insufficient data)
$^{13}C'$	0	—	None (insufficient data)
^{15}N	52	0.05 ± 1.04	None needed (< 0.5 ppm)

7.1.3 Completeness of resonance assignments (i)

The following table shows the completeness of the chemical shift assignments for the well-defined regions of the structure. The overall completeness is 48%, i.e. 316 atoms were assigned a chemical shift out of a possible 657. 0 out of 2 assigned methyl groups (LEU and VAL) were assigned stereospecifically.

	Total	^{1}H	^{13}C	^{15}N
Backbone	158/268 (59%)	106/107 (99%)	0/108 (0%)	52/53 (98%)
Sidechain	139/347 (40%)	139/205 (68%)	0/122 (0%)	0/20 (0%)

Continued on next page...

Continued from previous page...

	Total	^{1}H	^{13}C	^{15}N
Aromatic	19/42 (45%)	19/22 (86%)	0/20 (0%)	0/0 (—%)
Overall	316/657 (48%)	264/334 (79%)	0/250 (0%)	52/73 (71%)

The following table shows the completeness of the chemical shift assignments for the full structure. The overall completeness is 48%, i.e. 316 atoms were assigned a chemical shift out of a possible 664. 0 out of 2 assigned methyl groups (LEU and VAL) were assigned stereospecifically.

	Total	^{1}H	^{13}C	^{15}N
Backbone	158/273 (58%)	106/109 (97%)	0/110 (0%)	52/54 (96%)
Sidechain	139/349 (40%)	139/206 (67%)	0/123 (0%)	0/20 (0%)
Aromatic	19/42 (45%)	19/22 (86%)	0/20 (0%)	0/0 (—%)
Overall	316/664 (48%)	264/337 (78%)	0/253 (0%)	52/74 (70%)

7.1.4 Statistically unusual chemical shifts (i)

The following table lists the statistically unusual chemical shifts. These are statistical measures, and large deviations from the mean do not necessarily imply incorrect assignments. Molecules containing paramagnetic centres or hemes are expected to give rise to anomalous chemical shifts.

Mol	Chain	Res	Type	Atom	Shift, *ppm*	Expected range, *ppm*	Z-score
1	A	24	THR	HB	2.29	5.82 – 2.52	-5.7
1	A	2	LYS	HD2	0.41	2.76 – 0.46	-5.2

7.1.5 Random Coil Index (RCI) plots (i)

The image below reports *random coil index* values for the protein chains in the structure. The height of each bar gives a probability of a given residue to be disordered, as predicted from the available chemical shifts and the amino acid sequence. A value above 0.2 is an indication of significant predicted disorder. The colour of the bar shows whether the residue is in the well-defined core (black) or in the ill-defined residue ranges (cyan), as described in section 2 on ensemble composition.

Random coil index (RCI) for chain A:

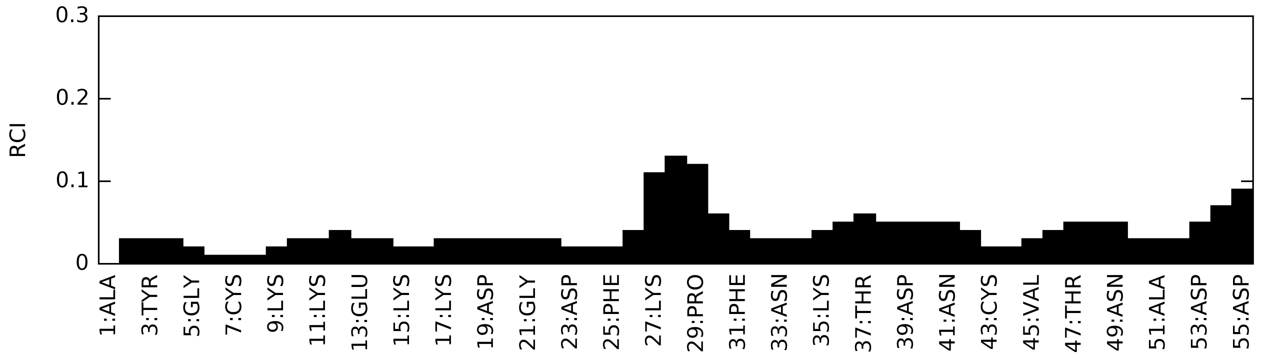
RCI
0.3
0.2
0.1
0
1:ALA
3:TYR
5:GLY
7:CYS
9:LYS
11:LYS
13:GLU
15:LYS
17:LYS
19:ASP
21:GLY
23:ASP
25:PHE
27:LYS
29:PRO
31:PHE
33:ASN
35:LYS
37:THR
39:ASP
41:ASN
43:CYS
45:VAL
47:THR
49:ASN
51:ALA
53:ASP
55:ASP
